# Lineage‐Specific Mesenchymal Stromal Cells Derived from Human iPSCs Showed Distinct Patterns in Transcriptomic Profile and Extracellular Vesicle Production

**DOI:** 10.1002/advs.202308975

**Published:** 2024-05-17

**Authors:** Tackla Winston, Yuanhui Song, Huaiyu Shi, Junhui Yang, Munther Alsudais, Maria I. Kontaridis, Yaoying Wu, Thomas R. Gaborski, Qinghe Meng, Robert N. Cooney, Zhen Ma

**Affiliations:** ^1^ Department of Biomedical & Chemical Engineering Syracuse University 329 Link Hall Syracuse NY 13244 USA; ^2^ BioInspired Institute for Materials and Living Systems Syracuse University 318 Bowne Hall Syracuse NY 13244 USA; ^3^ Departments of Biomedical and Chemical Engineering Rochester Institute of Technology One Lomb Memorial Drive Rochester NY 14623 USA; ^4^ Department of Biomedical Research and Translational Medicine Masonic Medical Research Institute 2150 Bleecker Street Utica NY 13501 USA; ^5^ Department of Medicine, Division of Cardiology, Beth Israel Deaconess Medical Center Harvard Medical School 330 Brookline Ave Boston MA 02215 USA; ^6^ Department of Biological Chemistry and Molecular Pharmacology Harvard Medical School Building C, 240 Longwood Ave Boston MA 02115 USA; ^7^ Department of Microbiology & Immunology SUNY Upstate Medical University 766 Irving Avenue Syracuse NY 13210 USA; ^8^ Department of Surgery State University of New York Upstate Medical University 750 East Adams Street Syracuse NY 13210 USA; ^9^ Sepsis Interdisciplinary Research Center State University of New York Upstate Medical University 766 Irving Avenue Syracuse NY 13210 USA; ^10^ Department of Biology Syracuse University 107 College Pl Syracuse NY 13210 USA

**Keywords:** extracellular vesicles, human iPSCs, mesenchymal stromal cells, transcriptomics

## Abstract

Over the past decades, mesenchymal stromal cells (MSCs) have been extensively investigated as a potential therapeutic cell source for the treatment of various disorders. Differentiation of MSCs from human induced pluripotent stem cells (iMSCs) has provided a scalable approach for the biomanufacturing of MSCs and related biological products. Although iMSCs shared typical MSC markers and functions as primary MSCs (pMSCs), there is a lack of lineage specificity in many iMSC differentiation protocols. Here, a stepwise hiPSC‐to‐iMSC differentiation method is employed via intermediate cell stages of neural crest and cytotrophoblast to generate lineage‐specific MSCs with varying differentiation efficiencies and gene expression. Through a comprehensive comparison between early developmental cell types (hiPSCs, neural crest, and cytotrophoblast), two lineage‐specific iMSCs, and six source‐specific pMSCs, are able to not only distinguish the transcriptomic differences between MSCs and early developmental cells, but also determine the transcriptomic similarities of iMSC subtypes to postnatal or perinatal pMSCs. Additionally, it is demonstrated that different iMSC subtypes and priming conditions affected EV production, exosomal protein expression, and cytokine cargo.

## Graphical abstract

Using a stepwise differentiation method, lineage‐specific induced mesenchymal stem cells (iMSCs) are derived from human induced pluripotent stem cells via intermediate cell stages of neural crest and cytotrophoblasts. A systematic comparison between two lineage‐specific iMSCs and six tissue‐specific primary MSCs shows that different MSC subtypes exhibit distinct transcriptomic profiles, anti‐inflammatory functions, and extracellular vesicle properties.

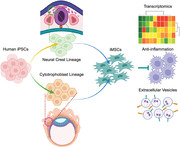

## 1 Introduction

Multipotent mesenchymal stromal cells (MSCs) have shown promises for tissue repair and regeneration, autoimmune diseases, and chronic disorders due to their therapeutic potentials in differentiation capacity, growth factor secretion, immunomodulation, and anti‐inflammatory responses.^[^ ^1^, ^2^, ^3^, ^4^ ^]^ Though MSCs can be found in various tissues, many tissue‐specific MSCs are not easily accessible for patient care due to limited availability of tissue source or the need for invasive surgical operation. Common MSC tissue sources, such as bone marrow and adipose tissue, can only yield ≈2% nucleated cells^[^ ^5^ ^]^ In addition, limited sources of perinatal tissues (umbilical cord, amniotic membrane, etc.) makes it difficult to obtain large amounts of MSCs from these tissues. More importantly, primary MSCs (pMSCs) collected from elderly donors suffer from fewer high‐quality cells, less therapeutic potency, and faster decline in proliferation and cell plasticity over repeated passages.^[^ ^6^, ^7^ ^]^ To overcome these challenges, researchers are exploring the differentiation of MSCs from pluripotent stem cells, such as human induced pluripotent stem cells (hiPSCs), as a limitless cell source for biomanufacturing purposes.^[^ ^8^, ^9^, ^10^ ^]^

pMSCs originating from different tissue sources show similar cell morphology, marker identity, and multi‐lineage differentiation capacity, but exhibit variations in growth rate, transcriptomic profile, secretome signature, anti‐inflammatory and immunomodulatory capacities.^[^ ^11^, ^12^, ^13^ ^]^ For example, a study reported that bone marrow‐derived pMSCs (BM‐pMSCs) and adipose tissue‐derived pMSCs (AD‐pMSCs) collected and paired from 14 healthy donors showed distinct gene expression patterns related to their tissue origin.^[^ ^13^ ^]^ However, it seems impossible to perform such paired comparative study between postnatal tissue‐derived MSCs (e.g., BM‐pMSCs) and perinatal tissue‐derived MSCs (e.g., amniotic membrane‐derived MSCs) from a single donor, which has led to contradictory results in the literature.^[^ ^14^, ^15^ ^]^ Such variability might originate from donor genetic and epigenetic background, tissue preparation techniques, cell culture and priming conditions, thus highlighting the need for standardization in both fundamental MSC biology and translational MSC therapy.

The differentiation of MSCs from pluripotent sources (iMSCs) offers opportunities for scalable biomanufacturing of these cells. It has been extensively reported that iMSCs exhibit similar multi‐differentiation potential and immunomodulation functions as pMSCs, but higher purity and potency due to their early developmental privilege.^[^ ^16^, ^17^, ^18^, ^19^ ^]^ The iMSC differentiation has been improving and optimizing over the years, and recent progress suggests that controlled lineage specification of iMSCs through defined stepwise differentiation processes gave rise to end‐stage iMSC subtypes with developmental lineage specificity. For example, iMSCs have been successfully differentiated through defined intermediate developmental stages of mesoderm,^[^ ^20^ ^]^ neural crest,^[^ ^21^, ^22^, ^23^ ^]^ and trophoblast‐like cells.^[^ ^24^ ^]^ A close comparison between iMSCs derived through mesoderm and through neuroepithelium indicated differences in paracrine signaling: mesoderm‐iMSCs had stronger HGF and EGF signaling for wound healing, while neuroepithelium‐iMSCs had stronger VEGF and FGF signaling for angiogenesis.^[^ ^25^ ^]^ Sharing a similar concept, lineage‐specific osteoprogenitor cells derived via the intermediate stages of paraxial mesoderm, lateral plate mesoderm and neural crest showed unique transcriptomic signatures associated with their developmental trajectories.^[^ ^26^ ^]^ Despite these early efforts, a systematic comparison of iMSCs from different lineages and pMSCs from different tissues is needed to define the developmental signatures of iMSCs and understand their commonalities and differences with pMSCs.

In this study, we differentiated two iMSC subtypes via two intermediate cell types of neural crest (NC‐iMSCs) and cytotrophoblast (CT‐iMSCs) using serum‐free chemical‐defined media. We also obtained six pMSCs from commercially available vendors, including bone marrow‐derived primary MSCs (BM‐pMSCs), adipose tissue‐derived primary MSCs (AD‐pMSCs), dental pulp‐derived primary MSCs (DP‐pMSCs), umbilical cord‐derived primary MSCs (UC‐pMSCs) chorionic villi‐derived primary MSCs (CV‐pMSCs), and chorionic plate‐derived primary MSCs (CP‐pMSCs). We performed a comprehensive comparison of lineage‐specific iMSCs and tissue‐specific pMSCs under the same serum‐free culture conditions. The results showed that iMSCs partially retained early developmental signatures compared to pMSC, meanwhile NC‐iMSCs and CT‐iMSCs had a closer transcriptomic pattern to postnatal and perinatal pMSCs, respectively. Transcriptomic analysis suggested that iMSCs and placental pMSCs had better potentials in EV biogenesis and trafficking than postnatal pMSCs. Furthermore, single‐cell RNA sequencing results showed heterogeneity in iMSC population, following a developmental trajectory from cycling pre‐MSCs, to MSCs, and then osteochondro‐progenitors. At the protein level, we further confirmed that CT‐iMSCs had slightly better exosomal EV production than NC‐iMSCs, making CT‐iMSCs a better candidate for therapeutic EV biomanufacturing.

## 2 Results

### 2.1 iMSC Differentiation via Neural Crest Lineage

**Figure 1:**
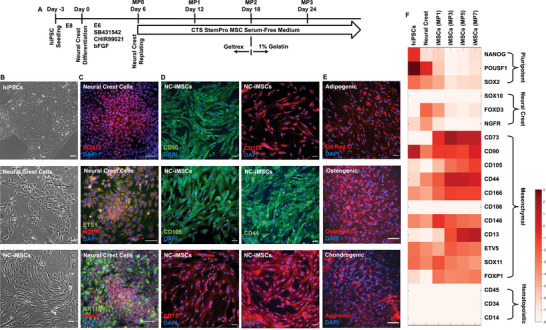
NC‐iMSC Differentiation. A) The differentiation protocol to derive iMSCs via an intermediate cell stage of neural crest. B) The phase‐contrast images showed the morphological changes from aggregated hiPSC colony to cuboidal‐shaped neural crest cells, and then spindle‐shaped iMSCs. C) The immunostaining results showed robust neural crest differentiation based on positive expression of SOX10, ETS1, NGFR, KRT19 and SNAI2. D) The immunostaining results showed successful NC‐iMSC differentiation based on positive expression of CD90, CD105, CD73, CD166, CD44, and CD146. E) NC‐iMSCs showed differentiation potentials into adipogenic, osteogenic and chondrogenic lineages. F) RT‐qPCR results showed cell fate transition during iMSC differentiation through the intermediate cell stages of neural crest cells.

There have been several studies demonstrating successful derivation of iMSCs via an intermediate stage of neural crest. During embryonic neurulation, neural crest cells are a transient cell type that develops at the border between neural plate and non‐neural ectoderm, delaminates via epithelial‐mesenchymal transition, and further differentiates into craniofacial musculoskeletal tissues. To differentiate the iMSCs with a neural crest signature, we first differentiated hiPSCs into neural crest cells by switching E8 media to E6 media supplemented with GSK3β inhibitor (CHIR99021), ALK inhibitor (SB431542), and bFGF (Day 0).^[^ ^21^, ^27^ ^]^ At Day 6, as Multipotent Passage #0 (MP0), the neural crest cells were then replated into the MSC serum‐free medium for further differentiation into iMSCs (**Figure** **1**A). We observed significant morphological changes from large hiPSC colonies transitioning into cuboidal‐shaped epithelial‐like cells (neural crest), and then MSC‐characteristic spindle‐like fibroblastic morphology starting at MP2 (Day 18), when the cells can be grown on 1% gelatin coating (Figure 1B).

We confirmed that hiPSCs highly expressed pluripotent markers of NANOG, OCT4 and SOX2 (Figure S1A, Supporting Information), and a high‐yield neural crest cell differentiation based on positive immunostaining of SOX10, neural growth factor receptor (NGFR), ETS1, cytokeratin‐19 (KRT19), and SNAI2 (Figure 1C). We were able to obtain robust neural crest‐to‐iMSC (NC‐iMSCs) differentiation based on positive immunostaining of typical MSC markers of CD90, CD105, CD73, CD166, CD44 and CD146 (Figure 1D), and negative of pluripotent markers of NANOG, OCT4 and SOX2 (Figure S1B, Supporting Information). Furthermore, our NC‐iMSCs showed differentiation potentials into adipogenic (oil red O), osteogenic (osteocalcin) and chondrogenic (aggrecan) lineages (Figure 1E). We confirmed that NC‐iMSCs can be grown on the plastic surface (6‐well plate) without any additional protein coating. To ensure our protocol can be robustly reproduced using another hiPSC line, we also differentiated NC‐iMSCs from the hiPSC line obtained from Yale University (Figure S2A, Supporting Information).

To further visualize the cell fate transition during NC‐iMSC differentiation, we performed gene expression profiling for pluripotency, neural crest, MSC, and hematopoietic stem cell (HSC) markers (Figure 1F). As expected, hiPSCs showed high gene expression of NANOG, OCT4, and SOX2. The neural crest‐related genes (FOXD3 and NGFR) showed a transient expression at the stages of neural crest cells (MP0) and early iMSC differentiation (MP1). Strong induction of typical MSC surface markers occurred around MP3 during the differentiation (CD73, CD105, CD13, CD44), while other MSC surface markers were also expressed in hiPSCs and neural crest cells (CD90, CD146, CD166). This result indicated that CD13 and CD73 might be good surface markers to identify and purify the NC‐iMSC population. Surprisingly, CD106, a gene highly associated with bone marrow‐derived MSCs, showed almost no expression from our NC‐iMSCs. We also selected three transcription factors (EVT5, SOX11 and FOXP1) that have been reported to relate to MSC identity, while only FOXP1 showed strong correlation with iMSCs. In addition, we confirmed no expression of HSC‐related genes (CD45, CD34 and CD14) from our NC‐iMSCs.

### 2.2 iMSC Differentiation via an Extraembryonic Lineage

The extraembryonic cells are typically derived from trophectoderm (TE), amniotic ectoderm (AME), and extraembryonic mesoderm (EEM) that give rise to the perinatal tissues to help sustain fetal growth and development. Early attempts to derive trophoblast‐like stem cells from hiPSCs were achieved based on ALK inhibition (A8301), FGF inhibition (PD173074) and BMP4 induction.^[^ ^28^ ^]^ However, recent studies indicated that induction of trophoblast differentiation from primed hiPSCs might result in cytotrophoblast cells present at post‐implantation stage, instead of trophoblast stem cells present at pre‐implantation stage.^[^ ^29^ ^]^ Herein, we differentiated our iMSCs from hiPSCs via cytotrophoblast lineage without converting hiPSCs to the naive stage. First, we determined whether it is necessary to inhibit FGF signaling during the cytotrophoblast differentiation, since a previous study reported successful iMSC differentiation via a trophoblast‐like stage without the use of FGF inhibitor.^[^ ^24^ ^]^ Meanwhile, we explored whether we could replace ALK inhibitor A8301 by SB431542 for cytotrophoblast differentiation, in order to keep it consistent with previous neural crest induction. Therefore, we tested the combination of BMP4 and PD173074 with different concentrations (0 µM, 0.1 µM, 0.25 µM, and 0.5 µM), together with either A8301 or SB431542, and then evaluated the gene expression associated with extraembryonic lineages (Figure S3, Supporting Information). Overall, the results demonstrated a high expression of cytotrophoblast genes (KRT7, TFAP2A, TFAP2C, PODXL), while relative low expression of extraembryonic mesoderm genes (RASIP1, LAMA4). We found there was no significant difference between A8301 and SB431542 for cytotrophoblast induction. We also confirmed that there was a critical need of FGF inhibition for higher expression of cytotrophoblast genes. Finally, we decided to use BMP4, SB431542 and low concentration of PD173074 (0.1 µM) for cytotrophoblast differentiation.

**Figure 2:**
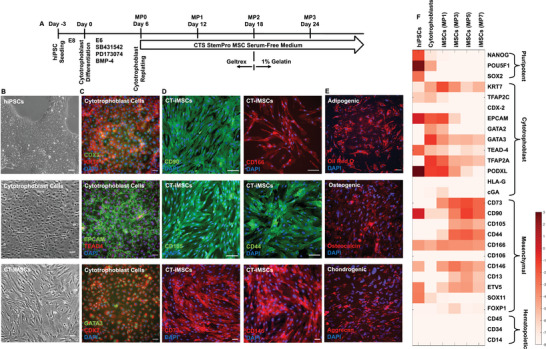
CT‐iMSC Differentiation. A) The differentiation protocol to derive iMSCs via an intermediate cell stage of cytotrophoblast cells. B) The phase‐contrast images showed the morphological changes from aggregated hiPSC colony to polygonal‐shaped cytotrophoblast cells, and then spindle‐shaped iMSCs. C) The immunostaining results showed robust cytotrophoblast differentiation based on positive expression of CDX2, KRT7, EPCAM, TEAD4, and GATA3. D) The immunostaining results showed successful CT‐iMSC differentiation based on positive expression of CD90, CD105, CD73, CD166, CD44, and CD146. E) CT‐iMSCs showed differentiation potentials into adipogenic, osteogenic and chondrogenic lineages. F) RT‐qPCR results showed cell fate transition during iMSC differentiation through the intermediate cell stages of cytotrophoblast cells.

To differentiate our iMSCs with an extraembryonic signature, we first differentiated hiPSCs into cytotrophoblast cells based on optimized protocol (Day 0). At Day 6, as Multipotent Passage #0 (MP0), the cytotrophoblast cells were then replated into the MSC serum‐free medium for further differentiation into iMSCs (**Figure** **2**A). We observed significant morphological changes from dense‐compacted hiPSC colonies to polygonal‐shaped cytotrophoblast‐like cells, and then MSC‐characteristic spindle‐like fibroblastic morphology starting at MP2 (Day 18), when the cells can be grown on 1% gelatin coating (Figure 2B). We confirmed successful cytotrophoblast differentiation based on positive immunostaining of CDX2, cytokeratin‐7 (KRT7), epithelial cellular adhesion molecule (EPCAM), TEAD4 and GATA3 (Figure 2C). We were able to obtain robust cytotrophoblast‐to‐iMSC (CT‐iMSCs) differentiation based on positive immunostaining of typical MSC markers of CD90, CD105, CD73, CD166, CD44 and CD146 (Figure 2D), and negative of pluripotent markers of NANOG, OCT4 and SOX2 (Figure S1C, Supporting Information). Furthermore, our CT‐iMSCs showed differentiation potentials into adipogenic (oil red O), osteogenic (osteocalcin) and chondrogenic (aggrecan) lineages (Figure 2E). nWe confirmed that CT‐iMSCs can be grown on the plastic surface (6‐well plate) without any additional protein coating. Similarly, our CT‐iMSC differentiation can be reproduced using the Yale hiPSC line (Figure S2B, Supporting Information).

Similar to NC‐iMSC differentiation, we also performed gene expression profiling for cell fate transition during CT‐iMSC differentiation (Figure 2F). The cells lost the expression of pluripotent markers (NANOG, OCT4, and SOX2) rapidly during the differentiation, and meanwhile early trophoblast markers (GATA2, GATA3, TEAD4, and TFAP2A) showed a transient expression at the stages of cytotrophoblast cells (MP0) and early iMSC differentiation (MP1). Our CT‐iMSCs showed a similar pattern of iMSC marker expression as NC‐iMSCs, further confirming that CD73, CD105, CD13, and CD44 were more exclusive to iMSCs in comparison to hiPSCs, neural crest, or cytotrophoblasts. However, CD146 and CD90 expression were significantly reduced at the cytotrophoblast stage, compared to the neural crest stage. Compared to NC‐iMSCs, CT‐iMSCs only showed robust expression of EVT5 as a key MSC transcription factor, but very low expression of SOX11 and FOXP1, which are highly related to germ layer differentiation. In addition, we also confirmed there was no expression of HSC‐related genes (CD45, CD34 and CD14) from the CT‐iMSCs.

### 2.3 Cell Heterogeneity in iMSC Development

To study how iMSCs emerged during two lineage‐specific differentiation processes, we performed flow cytometry to track iMSC population (CD73+, CD105+, CD90+, CD45‐) from MP0 to MP7 (Figure S4A, Supporting Information). For neural crest‐to‐iMSC differentiation, we observed that NC‐iMSC population rapidly increased within the first 12 days (Figure S4B, Supporting Information). At MP2, differentiation of CD90+ cells reached a plateau (≈80%), while CD73+ cells and CD105+ cells were only ≈60% and ≈30%, respectively, indicating that CD105 was expressed relatively late during the differentiation. For cytotrophoblast‐to‐iMSC differentiation, CT‐iMSC population increased at a slower pace compared to the NC‐iMSCs (Figure S4C, Supporting Information). At MP6 and MP7, it seemed that CD90+ and CD73+ cells were relatively stable at ∼80%, but CD105+ cells were still ramping up. Comparing the iMSC population at MP7 between two lineage‐specific differentiation, NC‐iMSCs had a higher yield in all three markers (≈90% of CD73+/CD90+/CD105+ cells) than the CT‐iMSCs (≈85% of CD73+/CD90+ cells, while ≈75% of CD105+ cells) (Figure S4D, Supporting Information). In addition, CD45+ cells were lower than 2% in average for both differentiation pathways.

**Figure 3:**
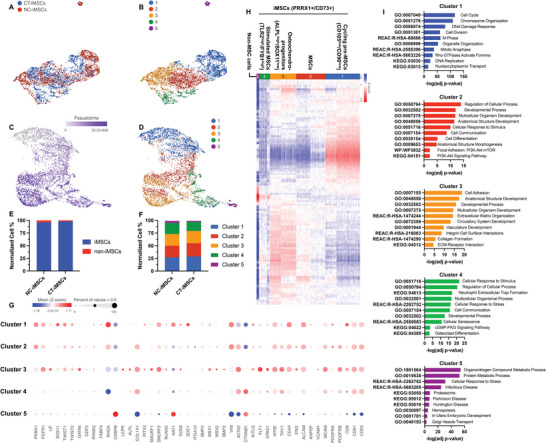
iMSC heterogeneity in development. A) UMAP projection of single cell RNA sequencing data from NC‐iMSCs and CT‐iMSCs, B) which was then re‐grouped into 5 cell clusters. C) Trajectory pseudo‐time analysis on iMSC heterogeneity showed D) a development branching between cluster 3 and cluster 4&5. E) Quantification of iMSCs versus non‐iMSCs and F) cell composition from 5 clusters showed comparable results between NC‐iMSCs and CT‐iMSCs. G) Dot plot showed differentially expressed genes associated with each cluster. H) Gene expression pattern of top 1000 most variable genes from annotated cell clusters: cycling pre‐MSCs, MSCs, osteochondro‐progenitors, stimulated MSCs and non‐MSCs. I) Gene ontology and pathway enrichment for all 5 cell clusters.

To better determine the iMSC development and heterogeneity, we performed single‐cell RNA sequencing (scRNAseq) on NC‐iMSCs and CT‐iMSCs to investigate the heterogeneity of the iMSC population. A total of 5088 cells were captured for NC‐iMSCs (n = 2865) and CT‐iMSCs (n = 2223). From uniform manifold approximation and projection (UMAP) plots, we observed that NC‐iMSCs and CT‐iMSCs overlapped with each other (**Figure** **3**A), and entire cell populations were divided into 5 clusters (Figure 3B) based on graph‐based clustering technique. From our gene expression projection on UMAPs, we observed cluster 1–4 highly expressed all MSC surface markers (CD44, D105, CD73 CD166 and CD90) and transcription factors (PRRX1, TWIST1, MSX1, SOX11, and GATA6) (Figure S5, Supporting Information). In addition, clusters 1–4 also expressed genes related to growth factor production (VEGF, FGF2, PDGFA, and EGF), while PGF expression seems to be low and more located to CT‐iMSC population. Cluster 1–4 showed high potential in osteogenic and chondrogenic differentiation (RUNX2, SOX9, and SDC1), but less in adipogenic potential (PPARG and FABP4). All these results indicated that cells from cluster 1–4 were iMSCs (≈ 95% of total cell population), while cells from cluster 5 were non‐iMSCs (only ≈5%) for both NC‐iMSCs and CT‐iMSCs (Figure 3E).

To identify cluster signature, we plotted top differentially expressed genes associated with each cluster in the UMAP (Figure S5, Supporting Information) and the dot plot (Figure 3G) for cell annotation. Similarly, we observed high expression of MSC markers from cluster 1–4, annotated as PRRX1+/CD73+ MSCs. Cluster 1 highly expressed cell cycle gene (CDK1), but relatively lower expression of MSC markers (CD90 and CD105), thus we annotate cluster 1 as cycling pre‐iMSCs. Cluster 2 highly expressed early chondrogenesis gene (ITGA10), while cluster 3 highly expressed osteogenesis gene (ALPL) but reduced expression of SOX11 compared to cluster 2, indicating a cell fate progression from cluster 2 (MSCs) to cluster 3 (early osteochondro‐progenitors). Cluster 4 and 5 expressed genes associated with cell stress and hemopoiesis (TLR2, FYB1, and SPI1). Due to the MSC identity associated with cluster 4, we believe this cluster represented the stimulated MSCs, while cells from cluster 5 were non‐MSC stromal cells. The composition percentile of these 5 cell clusters is comparable between NC‐iMSCs and CT‐iMSCs (Figure 3F). Next, we selected the top 1000 genes that were differentially expressed across these 5 clusters (Figure 3H) and performed gene and pathway enrichment analysis (Figure 3I). Cycling pre‐MSCs (cluster 1) were highly enriched in genes associated with cell cycle, cell division and DNA replication, while MSCs (cluster 2) and osteochondro‐progenitors (cluster 3) were highly enriched in genes associated with cell differentiation, tissue development, and cell‐ECM interaction. Stimulated MSCs (cluster 4) and non‐MSC stromal cells (cluster 5) were both enriched in genes associated with cell stress and cell senescence, while non‐MSC stromal cells expressed the genes associated with neural diseases and hemopoiesis.

We further performed pseudo‐time trajectory analysis on these cells using Monocle 3 to elucidate their expression patterns in an ordered differentiation path (Figure 3C). We observed that cycling pre‐MSCs were located at the beginning of the trajectory, indicating their earlier developmental stages. The cells progressed to the intermediate stage of MSCs, and then branched into either osteochondro‐progenitors (cluster 3) or stimulated cells with activated inflammatory responses (cluster 4 and cluster 5) (Figure 3D). We also profiled MSC/stromal‐related genes across the entire pseudo‐time. The gene expression of NT5E (CD73), CD44 and VIM were relatively stable across the pseudo‐time, while an increased expression of ENG (CD105) was observed at the later stages (Figure S6A, Supporting Information). Surprisingly, THY1 (CD90), POSTN, and ACTA2 fluctuated in their expression across the cell trajectory (Figure S6B, Supporting Information). For MSC transcription factors, PRRX1 and SOX11 decreased at the later stages, while SOX9 seems to increase, indicating the cells transitioning to an osteochondro‐progenitor fate (Figure S6C, Supporting Information).

### 2.4 Transcriptomic Comparison Between Early Developmental Cell Types and MSCs

We next performed bulk RNA sequencing (RNAseq) to investigate the impact of developmental lineage on iMSC properties and compare our iMSCs to the primary MSCs (pMSCs) isolated from different tissue sources (Table S1, Supporting Infromation). Different pMSCs, including bone marrow‐derived pMSCs (BM‐pMSCs), adipose tissue‐derived pMSCs (AD‐pMSCs), dental pulp‐derived pMSCs (DP‐pMSCs), umbilical cord‐derived pMSCs (UC‐pMSCs), chorionic villi‐derived pMSCs (CV‐pMSCs) and chorionic plate‐derived pMSCs (CP‐pMSCs), were cultured under the same condition as iMSCs using serum‐free media (Figure S7, Supporting Information) and used for RNA extraction at passage #3. The iMSCs (NC‐iMSCs and CT‐iMSCs) were sorted based on CD73 at MP7 for RNA extraction. In addition, we also include hiPSCs, neural crest cells and cytotrophoblast cells as early developmental cell types for RNAseq analysis (total 22 samples).

**Figure 4:**
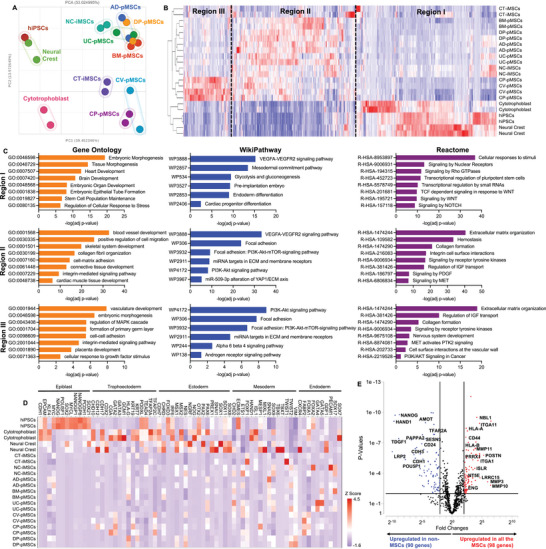
Transcriptomic profile of iMSC differentiation. A) Principal component analysis (PCA) plot showed separation between hiPSCs, intermediate cell types (neural crest and cytotrophoblast), lineage‐specific iMSCs and source‐specific pMSCs. B) Top 1000 high‐variance genes showed distinct gene expression pattern allocated into three regions. C) Gene ontology, WikiPathway and Reactome analysis showed distinct biological process and signaling pathway associated with the specific region. D) All 11 different cell types (hiPSCs, 2 intermediate cells, 2 iMSCs and 6 pMSCs) showed distinct gene expression associated with early embryonic development. E) Volcano plot showed differential gene expression between all MSCs (2 iMSCs and 6 pMSCs) and non‐MSC cells (hiPSCs, neural crest cells and cytotrophoblast cells).

After trimming down low‐quality reads, ≈ 24000 genes were used for the downstream analysis. Principal component analysis (PCA) showed clear separation between hiPSCs, neural crest cells, cytotrophoblast cells, and all the MSCs (Figure S8A, Supporting Information). Pearson's correlation matrix showed close correlation among early developmental cell types (hiPSCs, neural crest cells and cytotrophoblast cells) with CT‐iMSCs, while NC‐iMSCs were more closely correlated with AD‐pMSCs, BM‐pMSCs, and UC‐pMSCs (Figure S8B, Supporting Information). Next, we selected top 1000 genes with the highest variance from all 22 samples and re‐performed the PCA analysis, which showed a clear separation between two distinct MSC populations: one cluster of NC‐iMSCs, BM‐pMSCs, AD‐pMSCs and DP‐pMSCs, and the other cluster of CT‐iMSCs, CV‐pMSCs, and CP‐pMSCs (**Figure** **4**A). This indicated a close relationship between CT‐iMSCs differentiated with extraembryonic specificity and pMSCs derived from placental tissues.

To elucidate the gene expression pattern and sample correlation, we showed the top 1000 genes in a heatmap with hierarchical cluster analysis (Figure 4B). The segregation of these genes can be identified as three regions: region 1 of highly expressed genes for early developmental cell types, region 2 of highly expressed genes for all MSCs, and region 3 of highly expressed genes for placental pMSCs. Next, we performed a global analysis of biological processes (Gene Ontology) and signaling pathways (WikiPathway and Reactome Pathway) (Figure 4C). Enriched biological processes for region I highlighted early embryonic development and stem cell maintenance, while pathway analysis showed high enrichment in VEGF signaling and WNT signaling, which are associated with stem cell maintenance and germ layer differentiation. Region II and region III showed distinct GO terms associated with tissue development: region II for the development of skeletal tissues and connective tissues versus region III for the development of placenta and vasculature. However, pathway analysis showed similar enrichment between region II and III, highlighting extracellular matrix organization, focal adhesion, and PI3K‐AKT signaling.

With a particular focus on the lineage specificity of differentiated iMSCs, we selected the genes associated with early embryonic development (Figure 4D). As expected, hiPSCs highly expressed the genes associated with epiblasts (e.g., NANOG, POU5F1, SOX2, MYC) and cytotrophoblast cells highly expressed the genes associated with trophectoderm (e.g., GATA2, GATA3, TFAP2A, KRT7). Neural crest cells showed highly expressed genes from both ectoderm (SOX8, PAX2, NGRF) and mesoderm (MESP1, TBXT, MIXL1), indicating their developmental transition from neural tube to mesoderm. Both iMSCs and pMSCs showed relatively lower expression in these early developmental genes. Compared to postnatal pMSCs (BM‐pMSCs, AD‐pMSCs and DP‐pMSCs), perinatal pMSCs (UC‐pMSCs, CP‐pMSCs and CV‐pMSCs) showed more expression in this gene list due to their early developmental stages. CP‐pMSCs and CV‐pMSCs showed some level of expression in GATA2, KRT19 and TFAP2C, indicating their original source of perinatal tissues. UC‐pMSCs showed high expression of endoderm genes (GATA4, GATA5, and SOX17), indicating their developmental origin of hypoblast cells from primitive endoderm. For the iMSCs derived from different lineages, CT‐iMSCs and NC‐iMSCs were able to partially retain their developmental identity (e.g., GATA3 expression in CT‐iMSCs, NES expression in NC‐iMSCs), but more differentiated to a mesoderm lineage.

Last, we combined all the MSCs and compared their gene expression to early developmental cell types using volcano plots thresholding at p‐values < 0.01 and fold changes > 4. The genes upregulated in hiPSCs were the markers associated with pluripotency and reprogramming (NANOG, POU5F1, SPINT1, TERF1), while the genes upregulated in MSCs were found as typical MSC markers (VIM, NT5E, CD44) (Figure S8C, Supporting Information). By comparing neural crest cells and cytotrophoblast cells, we observed that high gene expression in cytotrophoblast cells were associated with epithelial development (EGFR, ANKS1A, ZFHX3) and immunomodulatory properties (VTCN1, ANXA1), while high gene expression in neural crest cells were associated with neural development (TUBB2A, ENO2, VGF) (Figure S8D, Supporting Information). Compared to neural crest or cytotrophoblast cells, MSCs showed significant upregulation in genes associated with cell‐ECM interactions (ITGBL1, ECM1, MMP1, COL3A1, and COL8A1), osteogenic differentiation (RUNX1, PITX1, and LRRC15), and immunomodulation (ANXA1, DLC1, and INHBA) (Figure S8E–F, Supporting Information). Last, we compared all the MSCs (six pMSCs and two iMSCs) to all the non‐MSCs (hiPSCs, neural crest and cytotrophoblast). Non‐MSCs upregulated genes in early embryonic and placental development (NANOG, POU5F1, TFAR2A, HAND1, PAPPA2, CDH1, and CDH3), while MSCs upregulated MSC‐typical genes (CD44, NT5E, POSTN, ENG) and ECM‐related genes (COL3A1, COL6A1, MMP3, MMP10, and MMP11) (Figure 4E). We performed a network analysis on 98 genes that were upregulated in all the MSC subtypes (Figure S9, Supporting Information), and identified PRRX1, as the key transcription factor related to many MSC‐related functions, such as MSC identify (NT5E, CD44), ECM deposition and remodeling (collagens, MMPs), and growth factor signaling (NRP1, PDGFRA, IGFBP7, ISLR).

### 2.5 Transcriptomic and Functional Comparison Between iMSCs and pMSCs

**Figure 5:**
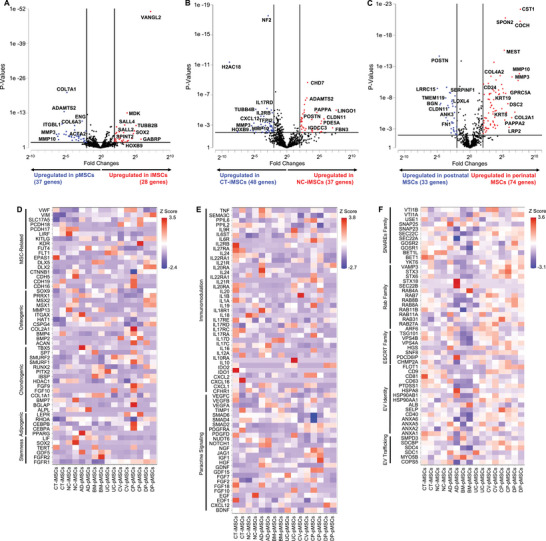
Transcriptomic comparison between lineage‐specific iMSCs and source‐specific pMSCs. Volcano plots showed differential gene expression for A) 6 pMSCs versus 2 iMSCs, B) CT‐iMSCs versus NC‐iMSCs, and C) postnatal MSCs (AD‐pMSCs, BM‐pMSCs, DP‐pMSCs, NC‐iMSCs) versus perinatal MSCs (CV‐pMSCs, CP‐pMSCs, UC‐pMSCs, CT‐iMSCs). All 8 different MSC types (2 iMSCs and 6 pMSCs) showed distinct gene expression associated with D) stemness and tri‐lineage differentiation potential, E) paracrine signaling and immunomodulatory potential, and F) EV production.

Focusing on the MSC populations, we first compared two lineage‐specific iMSCs and six tissue‐specific pMSCs (**Figure** **5**A). Key genes associated with early embryonic and neural development (VANGL2, MDK, TUBB2B, SOX2, SPINT2, GABRP) were upregulated in the iMSCs, indicating their early developmental stages compared to the pMSCs. The genes upregulated in the pMSCs were more associated with cell‐ECM interactions (ITGBL1, COL7A1, COL6A3, MMP3 and MMP10). Surprisingly, pMSCs showed higher expression of gene ENG (CD105) than iMSCs, which indicates CD105 might be a late marker for MSC differentiation. Next, we compared NC‐iMSCs and CT‐iMSCs (Figure 5B), and found that upregulation of genes in NC‐iMSCs associated with musculoskeletal development (POSTN, CHD7, FBN3, ADAMTS1 and CLDN11) and upregulation of genes in CT‐iMSCs associated with tumor suppressor (NF2, TFPI2, H2AC18), immunomodulation (CXCL12, IL2RB, and IL17RD) and ECM remodeling (MMP3 and MMP10).

We grouped BM‐pMSCs, AD‐pMSCs, DP‐pMSCs together with NC‐iMSCs as postnatal tissue MSCs, and UC‐pMSCs, CP‐pMSCs, CV‐pMSCs together with CT‐iMSCs as perinatal tissue MSCs. For the postnatal tissue MSC group, 269 genes were shared by these four MSC subtypes (Figure S10A, Supporting Information). Since neural crest cells give rise to craniofacial musculoskeletal tissues during development, NC‐iMSCs shared more genes with DP‐pMSCs than AD‐pMSCs and BM‐pMSCs. For the perinatal tissue MSC group, 229 genes were shared by these four MSC subtypes (Figure S10B, Supporting Information). Comparing the gene upregulation in these two groups, we found that genes associated with placental development and maternal immune compatibility (PAPPA2, COCH, MEST, SPON2, and CD24) were upregulated in perinatal tissue MSCs, while genes associated with musculoskeletal development (POSTN, CLDN11, TMEM119, BGN, FN1, LRRC15) were upregulated in postnatal tissue MSCs (Figure 5C). By comparing NC‐iMSCs to the other postnatal tissue pMSCs, genes associated with neural development (TUBB2B, SOX2, GATA3, KRT8) were upregulated due to their neural epithelium signatures (Figure S10C, Supporting Information). By comparing CT‐iMSCs to the other perinatal tissue pMSCs, genes associated with early embryonic development (VANGL2, DPPA4, ITM2C, CDH1) and placental cadherin (CDH3) were upregulated due to their early developmental stage (Figure S10D, Supporting Information).

We also selected the genes‐of‐interest associated with MSC functions (stemness, tri‐lineage differentiation, angiogenesis, paracrine signaling, immunomodulation, extracellular vesicle (EV) biogenesis and trafficking) and investigated the expression level for all the MSC subtypes. Overall, we found that all MSC subtypes had similar potential in tri‐lineage differentiation, while CP‐pMSCs and AD‐pMSCs showed a slight favor to the adipogenic differentiation (Figure 5D). All MSCs, including two iMSC subtypes, showed a great potential for immunomodulatory functions and growth factor signaling based on our gene set (Figure 5E). Focusing on EV biogenesis and trafficking (ESCRT, Rab, and SNARE protein families), we found iMSCs (CT‐iMSCs and NC‐iMSCs) and perinatal pMSCs showed greater potential in EV production than AD‐pMSCs and BM‐pMSCs (Figure 5F).

To compare the tri‐lineage differentiation of different MSC subtypes, we induced adipogenic, osteogenic, and chondrogenic differentiation on each MSC subtype (Figure S11, Supporting Information). All MSCs elevated lineage‐specific gene expression under specific induction media: CEBPA and PPARG for adipogenesis, RUNX2 and SPP1 for osteogenesis, and SOX9 and ACAN for chondrogenesis. We found no significant difference across different MSC subtypes, indicating that all MSC subtypes shared similar abilities to be induced for lineage‐specific differentiation.

To compare the anti‐inflammatory function of different MSC subtypes, we set up a transwell co‐culture experiment between RAW264.7 cells and each MSC subtype (Figure S12, Supporting Information). Co‐cultures were treated with Lipopolysaccharide (LPS), and several genes associated with inflammatory responses were measured from RAW264.7 cells via RT‐qPCR. The gene expression level was normalized to the negative control group (single‐cultured RAW264.7 cells without LPS treatment). We observed that positive control group (single‐cultured RAW264.7 cells with LPS treatment) showed a dominantly high expression of cytokine genes (TNFα, IL1β, IL6, IL10), which can be attenuated by MSC co‐culture. More importantly, the downregulation of pro‐inflammatory genes (TNFα, IL1β, and IL6) was more prominent from the co‐culture with iMSCs than the ones with UC/DP/CP‐pMSCs. More importantly, RAW246.7 cells co‐cultured with iMSCs showed significantly higher expression of ARG1 gene than positive control and pMSC co‐culture groups, indicating that iMSCs facilitated RAW246.7 cells to transition to an anti‐inflammatory phenotype.

### 2.6 Extracellular Vesicle (EV) Production from Lineage‐Specific iMSCs

**Figure 6:**
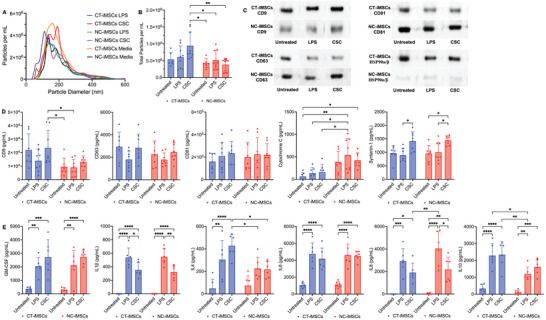
Production of extracellular vesicles (EVs) from iMSCs. A) Nanoparticle tracking analysis showed comparable particle size distribution for purified iMSC‐produced EVs under different priming conditions, but B) higher EV concentration from CSC‐primed CT‐iMSCs than the NC‐iMSCs. C) Western blot experiments showed different expression of exosomal proteins between CT‐iMSCs and NC‐iMSCs. D) Luminex assay of exosome biomarkers showed slightly enhanced expression of exosomal tetraspanin proteins (CD9) and significantly reduced expression of apoptotic cell bodies protein (cytochrome c) from CSC‐primed CT‐iMSCs. E) Luminex assay of human cytokines showed both priming conditions could significantly enhance the cytokine concentration in iMSC‐EVs, while EVs produced from CSC‐primed CT‐iMSCs had higher anti‐inflammatory cytokine expression (IL4 and IL10) than EVs from NC‐iMSCs. Statistics: two‐way ANOVA with post‐hoc Tukey test corrected for multiple comparison and *p*<0.05 is considered as significant difference (n ≥ 6).

EVs produced by MSCs are highlighted for their multifaceted therapeutic potentials via several simultaneous actions: inhibit inflammation, modulate immune responses, reduce cell apoptosis, and enhance tissue repair and regeneration.^[^ ^30^, ^31^, ^32^ ^]^ With a particular interest in EV biomanufacturing from iMSCs, we primed both NC‐iMSCs and CT‐iMSCs using either LPS or Cell Stimulation Cocktail (CSC). The CSC is a cocktail of phorbol 12‐myristate 13‐acetate (PMA) and ionomycin, which could activate many cell types to produce cytokines. We collected and purified the small EVs (sEVs) from cell culture media. Nanoparticle tracking analysis (NTA) showed the size distribution of sEVs within the range of 50 – 400 nm with the majority of the particles smaller than 200 nm (**Figure** **6**A). We also observed a higher sEV concentration from CT‐iMSCs primed with CSC than the NC‐iMSCs (Figure 6B). To confirm exosome‐identity, we performed western blot on our sEVs isolated from both CT‐iMSCs and NC‐iMSCs under different priming conditions (Figure 6C; Figure S13, Supporting Information). The sEVs from both CT‐iMSCs and NC‐iMSCs had robust comparable expression of CD63 and CD81, while sEV from CT‐iMSCs had higher expression of CD9 than the ones from NC‐iMSCs. More surprisingly, the sEVs from NC‐iMSCs lacked the expression of HSP90α/β, which was highly expressed by the sEVs from CT‐iMSCs. A recent study showed that HSP90 mediates multivesicular bodies (MVB)‐to‐plasma‐membrane fusion, indicating that HSP90 proteins promote exosome release.^[^ ^33^ ^]^

To quantitatively compare the sEVs production for different priming conditions, we performed a Luminex assay on exosome biomarkers (Figure 6D). Overall, the expression level of exosomal tetraspanin markers were comparable between CT‐iMSCs and NC‐iMSCs (CD63, CD81, syntenin‐1). Similar to the western blot results, CD9 in the sEV from CSC‐primed CT‐iMSCs was higher than the ones from untreated NC‐iMSCs or LPS‐primed NC‐iMSCs. Syntenin‐1 was recently identified as the highest consistently abundant protein in the exosomes from different cellular origins, with potential utility as a putative universal biomarker candidate for exosomes.^[^ ^34^ ^]^ We found a higher syntenin‐1 expression from CSC‐primed iMSCs than LPS‐primed iMSCs for both iMSC subtypes, suggesting the expression level of syntenin‐1 might depend on different priming conditions. Cytochrome c, which is related to apoptotic cell bodies, is generally used as a negative marker for exosomes.^[^ ^35^ ^]^ We found minimal expression of cytochrome c from CT‐iMSCs produced sEVs, while NC‐iMSCs produced sEVs were significantly higher for all conditions, indicating that sEVs from NC‐iMSCs might include a higher content of non‐exosomal vesicles.

With a particular interest on the anti‐inflammatory properties of iMSC‐sEV, we also performed a Luminex assay to measure the cytokine level within the sEV cargo (Figure 6E). First, we were not able to detect IFNγ, IL2, IL5 and TNFα, which are generally recognized as pro‐inflammatory proteins. The production of other cytokines, including both pro‐inflammatory proteins (IL1β, IL6, IL8) and anti‐inflammatory proteins (GM‐CSF, IL4, IL10) can be significantly enhanced by either LPS or CSC treatment. To compare different priming conditions, we observed a reduction of pro‐inflammatory proteins (IL1β and IL8) in the sEV from CSC‐primed iMSCs compared to LPS‐primed iMSCs. To compare different iMSC subtypes, we observed that the sEV from CT‐iMSCs, particularly under the CSC priming condition, contained higher concentration of anti‐inflammatory proteins (IL4 and IL10) than the ones from NC‐iMSCs. Overall, CT‐iMSCs under CSC priming condition produced the sEV in higher quantity and with stronger anti‐inflammatory properties than NC‐iMSCs, indicating that CT‐iMSCs plus CSC priming condition might enable a potential cell source for therapeutic EV biomanufacturing applications.

## 3 Discussion

### 3.1 Development of iMSC Differentiation with Lineage Specificity

In this study, we have successfully demonstrated differentiation of iMSCs from monolayer hiPSCs under serum‐free condition. Early attempts to different iMSC from human pluripotent cells (hESCs and hiPSCs) primarily relied on the formation and growth of embryoid bodies (EBs) on different substrates.^[^ ^17^, ^36^, ^37^, ^38^, ^39^, ^40^ ^]^ In these approaches, the outgrowing cells from the EBs were harvested through mechanical scraping or trypsinization, and then replated back into MSC‐defined growth media until the cells developed MSC characteristics. Though recent efforts have moved away from the need for EB formation, 3D bioreactor platforms are being developed to generate MSC spheroids from EBs in suspension without the need for repeated passaging.^[^ ^41^ ^]^ Progression was made in deriving iMSCs from monolayer hiPSCs based on temporal inhibition of ALK signaling using SB431542, followed by continuous culture in MSC‐defined media.^[^ ^42^, ^43^, ^44^, ^45^ ^]^ Although these studies have demonstrated successful iMSC induction, the intermediate stages of differentiation were less characterized and defined.

In addition to advancements in differentiation strategies, significant effort has been dedicated to in‐depth characterization of intermediate cell stages and cell‐fate transitions that underlie the differentiation process. Several studies, including our own, have demonstrated successful differentiation of iMSCs through a neural crest cell lineage.^[^ ^21^, ^22^, ^23^ ^]^ Meanwhile, inhibition of IKK/NF‐κB signaling, or activation of Activin/BMP signaling has been shown to induce iMSC differentiation via mesoderm lineage.^[^ ^20^, ^46^, ^47^ ^]^ iMSCs were also derived from trophoblast‐like cells induced from hESCs using BMP4 and ALK inhibitor.^[^ ^24^ ^]^ Despite differences in intermediate stages, these iMSCs exhibited MSC‐like immunophenotype, plastic adherent ability, and multipotent differentiation capacity, meeting the minimal criteria defined by the International Society for Cellular Therapy. In our work, we successfully induced iMSC differentiation via two different intermediate cell stages, neural crest and cytotrophoblast, using a serum‐free condition. Although both intermediate cell types require ALK inhibition, neural crest differentiation relies on WNT and FGF signaling, while cytotrophoblast differentiation relies on BMP signaling but FGF inhibition. Although iMSCs derived from different intermediate cells showed many similarities, differences were observed in yield, osteogenic potential, and marker expression.

### 3.2 Systematic Comparison of MSCs from Different Origins

After initial isolation from bone marrow, MSCs have been found in various tissues, with each subtype exhibiting source‐specific cell characteristics due to their local tissue microenvironment. A paired comparison between BM‐pMSCs and AD‐pMSCs from the same healthy donors indicated that AD‐pMSCs exhibited stronger immunosuppression properties and lower immunogenicity than BM‐pMSCs.^[^ ^13^ ^]^ Similarly, a paired comparison between placenta‐derived pMSCs and UC‐pMSCs from the same donors showed differences in immunomodulatory properties: placental pMSCs were more effective in inhibition of dendritic cells, while UC‐MSCs were more effective in inhibition of T cells.^[^ ^48^ ^]^ In general, there has been strong consensus that perinatal tissue‐derived pMSCs (amnion, chorion, placenta, umbilical cord, Wharton's Jelly, cord blood) have strong immunomodulatory properties due to immune tolerance to prevent fetal rejection during pregnancy.^[^ ^49^, ^50^, ^51^, ^52^ ^]^

Early comparisons between iMSCs and pMSCs focused on functional similarities of iMSCs as an alternative MSC source for therapeutic solutions.^[^ ^53^, ^54^ ^]^ More recently, comprehensive comparisons between iMSCs and pMSCs aimed to elucidate the distinct profiles for different MSC subtypes. Several reports indicated a reduction in adipogenic potential in iMSCs compared to BM‐pMSCs.^[^ ^42^, ^55^, ^56^ ^]^ In a study based on both transcriptomic and proteomic analysis, the top enriched biological processes found in iMSCs over BM‐pMSCs were related to embryo and neural development.^[^ ^57^ ^]^ In our study, we compared the transcriptomic profiles of MSCs to early development cells (hiPSCs, neural crest and cytotrophoblast cells), and found that MSCs were enriched in the genes related to osteogenesis, immunomodulation, and cell‐ECM interaction. Early developmental cells were enriched in VEGF and WNT signaling, while MSCs were enriched in FAK and AKT signaling. When comparing iMSCs and pMSCs, we observed that iMSCs retained early developmental characteristics, while pMSCs showed a stronger association with cell‐ECM interactions. On the transcriptomic level, CT‐iMSCs showed strong potential for immunomodulatory functions and EV biogenesis, which was confirmed by protein level analysis on the EVs produced from both iMSC subtypes. EVs produced from CT‐iMSCs showed higher expression of exosomal proteins (CD9 and HSP90), as well as immunosuppressive cytokine cargos (IL4 and IL10), compared to the EVs produced from NC‐iMSCs.

### 3.3 MSC‐Specific Transcription Factors

Although maintenance and self‐renewal of MSCs have been well established, it remains to be determined which transcription factors are critical in regulation and maintenance of MSC identify. Most previous studies have focused on investigating key transcriptional factors in regulating MSC differentiation into specific lineages, such as master transcription factors for osteogenic differentiation (RUNX2 and OSX), chondrogenic differentiation (SOX9 and FOXO3A), and adipogenic differentiation (PPARγ and EBF1).^[^ ^58^ ^]^ Early work on BM‐pMSCs identified nine transcription factors, including ETV1, ETV5, FOXP1, GATA6, HMGA2, SIM2 and SOX11, involved in self‐renewal and stemness of MSCs.^[^ ^59^ ^]^ More recently, MSX2 and TWST1 were found to play a critical role in initiating and accelerating the molecular program that led to iMSC differentiation via an intermediate cell stage of neural crest.^[^ ^60^ ^]^

From our scRNAseq data, we found that high expression of key transcription factors of MSX1, TWIST1, GATA6 genes was present in the iMSCs, which is consistent with previous literature. Expression of SOX11 was lower than the other transcription factors and decreased as MSCs progressed to the later stages based on pseudo‐time trajectory analysis, suggesting that SOX11 might initiate early transcriptional activity for MSC fate determination. Moreover, we identified a transcriptional factor PRRX1, which was highly involved in MSC identify (NT5E, CD44), signaling (WNT, PDGF), and ECM interactions. PRRX1 is known to regulate cancer metastasis by enhancing epithelia‐mesenchymal transition of cancer cells through the TGFβ, WNT, and NOTCH signaling pathways.^[^ ^61^ ^]^ It has also been recognized as an important factor for organogenesis of mesenchymal tissues and vascular structures during development.^[^ ^62^, ^63^ ^]^ Recently, PRRX1 was found to play a crucial role in tissue homeostasis for bone, white adipose, and dermal tissues in adult mice.^[^ ^64^ ^]^ This study showed RPPX1+ cells exhibited surface markers of CD29+, CD130+, CD31−, CD45−, but low expression of CD105, indicating that activation of CD105 expression in iMSCs might rely on additional transcription factors or endogenous signaling. While our findings are limited to the transcriptomic level, future studies are encouraged to investigate PRRX1 as a critical transcription factor and its related signaling activities for iMSC fate decision during development and pMSC maintenance in adulthood.

### 3.4 Differentiation of iMSCs via Extraembryonic Lineages

A previous study showed that iMSCs can be differentiated from hPSCs through trophoblast‐like cells using BMP4 and ALK inhibitor A8301 without PD173074.^[^ ^24^ ^]^ Our work showed the importance of PD173074 in promoting cytotrophoblast differentiation, which was consistent with previous reports.^[^ ^28^, ^65^, ^66^, ^67^ ^]^ However, our work is also limited to the use of primed hiPSCs for trophectoderm induction. To improve the differentiation of trophoblasts, it may be necessary to convert primed hiPSCs to the naïve stage, as previous studies have shown that TSCs derived from naïve hPSCs exhibit key features of pre‐implantation trophectoderm.^[^ ^29^, ^68^, ^69^, ^70^ ^]^ Further optimization of the differentiation protocol using naïve hiPSCs can enhance our understanding of the molecular mechanisms regulating trophoblast differentiation and lead to the development of more efficient and effective iMSC differentiation strategies.

Perinatal tissues are rich sources of pMSCs, which can be isolated from amniotic fluid (AF), amniotic membrane (AM), cord blood (CB), umbilical cord (UC), and placenta. Importantly, many cell types from these extraembryonic lineages play an essential role in maternal‐fetal immune tolerance during early embryo and fetal development. For instance, extravillous trophoblasts present HLA‐C, HLA‐E and HLA‐G to modulate maternal NK cells and T cells, thus balancing immune tolerance and antiviral immunity at the maternal–fetal interface.^[^ ^71^ ^]^ Our initial results on iMSC‐EVs indicated that EVs produced by CT‐iMSCs had better immunosuppressive capacity than those produced by NC‐iMSCs. Currently, protocols to generate different extraembryonic cells from hPSCs are still being optimized. Future study could focus on deriving iMSCs from these specific lineages to further elucidate the similarities and differences of extraembryonic iMSC subtypes, particularly in terms of their immunomodulatory and anti‐inflammatory properties.

### 3.5 Therapeutic Potential of Lineage‐Specific iMSCs

Currently, iMSCs have been reported to reduce ischemia and inflammation in various animal disease models, such as myocardial infarction, lower limb ischemia, inflammatory bowel disease, and acute lung injury.^[^ ^72^, ^73^, ^74^, ^75^, ^76^, ^77^ ^]^ Compared with tissue‐derived pMSC, iMSC closely resemble their primary counterparts in morphology, immunophenotype, and tri‐lineage differentiation capacity, while showing stronger regeneration ability in animal models. In 2016, Cynata Therapeutics from Australia launched the world's first trial of an allogeneic iMSCs for the treatment of steroid resistant acute graft‐versus‐host disease (GVHD), and now is advancing to Phase II trials for COVID‐19 and GVHD, and Phase III trials for osteoarthritis.^[^ ^78^, ^79^ ^]^ Our transcriptomic profiling of lineage‐specific iMSC subtypes suggests that iMSCs with different developmental signature can be designed for distinct therapeutic purposes. NC‐iMSCs with enriched transcriptomics toward musculoskeletal tissue development might be more suitable for osteoarthritis or bone defect repairing, while CT‐iMSCs with enriched transcriptomics toward immune tolerance might be more suitable for anti‐inflammatory applications. Our ongoing study also demonstrated the effectiveness of iMSC‐EVs in attenuating the inflammation in a murine acute lung injury model. Therefore, iMSCs enable a scalable source for “off‐the‐shelf” cell products under Good Manufacturing Practice (GMP) procedures for future therapeutic applications to treat complex and multifactorial diseases.

## 4 Conclusion

Using a stepwise differentiation method, this study generated lineage‐specific iMSCs from human induced pluripotent stem cells (iPSCs) via intermediate cell stages of neural crest and cytotrophoblast. We compared the transcriptomic profiles of early developmental cell types, two lineage‐specific iMSCs, and six source‐specific pMSCs, revealing that MSCs were enriched in genes related to osteogenesis, immunomodulation, and cell‐ECM interaction. NC‐iMSCs had a higher MSC purity and stronger osteogenic differentiation potential than CT‐iMSCs. However, CT‐iMSCs had better EV production and immunomodulatory function than NC‐iMSCs, making CT‐iMSCs a better candidate for therapeutic EV biomanufacturing. This study demonstrated that different iMSC subtypes and priming conditions affected EV production, exosomal protein expression, and cytokine cargo, highlighting the importance of generating lineage‐specific MSCs to improve their therapeutic potential.

## 5 Experimental Section

### iMSC Differentiation

The WTC hiPSC line was obtained from the Conklin lab at the Gladstone Institute of Cardiovascular Disease, and Yale hiPSC line was obtained from Kontaridis lab at the Masonic Medical Research Institute. The hiPSCs were plated at a density of 2.5 × 10^4^ cells cm^−2^ on Geltrex‐coated 6‐well plates in the Essential 8 (E8) media (*Life Technologies, Ca# A1517001*) supplemented with 10 µM Y‐27632 (*Biovision, Ca# 1784*). Growth factor reduced Geltrex (*Life Technologies, Ca# A1413302*) diluted for surface coating was prepared by thawing 5 mL of original Geltrex gel into 495 mL cold DMEM/F12 (*Life Technologies, Ca# 11320033*). The hiPSCs were maintained in the E8 media, and media was refreshed every day.

For NC‐iMSC differentiation, the hiPSCs were first treated for neural crest induction with 10 ng mL^−1^ bFGF (*R&D Systems Ca# 233‐FB*), 4 µM SB431542 (*Stemgent, Ca# 04‐0010‐10*), and 4 µM CHIR99021 (*Stemgent, Ca# 04–2004*) in Essential 6 (E6) media (*Life Technologies, Ca# A1516401*). The differentiation medium was changed daily for the next 5 days. On Day 6, the cells were plated as ‘*Multipotent Passage 0′* (**MP0**) on Geltrex‐coated 6‐well plates in serum‐free MSC culture medium (*CTS StemPro MSC SFM, Life Technologies, Ca# A1033201*) at a density of 4 × 10^4^ cells cm^−2^. Every 6 days, the cells were re‐plated at a density of 2 × 10^4^ cells cm^−2^ for MP1 – MP7. Starting from MP3, surface coating was switched from Geltrex to 1% Gelatin (*Life technologies, Ca# S006100*) to support iMSCs adhesion and growth.

For CT‐iMSC differentiation, the hiPSCs were first treated for cytotrophoblast induction with 10 ng mL^−1^ BMP4 (R&D Systems Ca# 314‐BP), 4 µM SB431542 (*Stemgent, Ca# 04‐0010‐10*), and 0.1 µM PD173074 (Stemcell technologies, 72164) in Essential 6 (E6) media (*Life Technologies, Ca# A1516401*). The differentiation medium was changed daily for the next 5 days. On Day 6, the cells were plated as ‘*Multipotent Passage 0′* (MP0) on Geltrex‐coated 6‐well plates in serum‐free MSC culture media (*CTS StemPro MSC SFM, Life Technologies, Ca# A1033201*) at a density of 4 × 10^4^ cells cm^−2^. Every 6 days, the cells were re‐plated at a density of 3 × 10^4^ cells cm^−2^ for MP1 – MP7. Starting from MP3, surface coating was switched from Geltrex to 1% Gelatin (*Life technologies, Ca# S006100*) to support iMSCs adhesion and growth.

### Primary MSC Culture

Six different primary MSCs were purchased from commercially available vendors (Table S1, Supporting Information), including bone marrow‐derived primary MSCs (BM‐pMSCs), adipose tissue‐derived primary MSCs (AD‐pMSCs), dental pulp‐derived primary MSCs (DP‐pMSCs), umbilical cord‐derived primary MSCs (UC‐pMSCs) chorionic villi‐derived primary MSCs (CV‐pMSCs), and chorionic plate‐derived primary MSCs (CP‐pMSCs). The primary MSCs were plated on 1% Gelatin‐coated 6‐well plates in serum‐free MSC culture media (*CTS StemPro MSC SFM, Life Technologies, Ca# A1033201*) at a density of 1 × 10^4^ cells cm^−2^. Every 6 days, the cells were replated at a density of 1 × 10^4^ cells cm^−2^ in serum‐free MSC culture medium.

### Co‐culture of MSCs and RAW 264.7 Cells

RAW 264.7 cells were cultured in DMEM supplemented with 10% fetal bovine serum (Gibco) and 1% penicillin/streptomycin at 37 °C with 5% CO_2_. In transwell co‐culture experiment, RAW264.7 cells were plated in the lower chamber at 5×10^4^ cells well^−1^. Different subtypes of MSCs were seeded at the density of 5×10^4^ cells/insert in the upper compartment of 24‐transwell plates (Corning, USA) (3.0 µm pore polycarbonate membrane). After co‐culture for 24 hours, the cells were treated with lipopolysaccharide (LPS) (1 µg ml^−1^) for another 24 hours. Next, RAW 264.7 cells were harvested for subsequent RT‐qPCR analysis of inflammation‐related genes. Single‐cultured RAW264.7 cells with and without LPS treatment were used as positive and negative controls, respectively. ‐∆∆Ct was calculated relative to the negative control.

### Immunostaining and Fluorescent Microscopy

The cells were fixed with 4% (vol/vol) paraformaldehyde (PFA) for 15 minutes, permeabilized with 0.2% triton solution for 5 minutes, and blocked with 2% bovine serum albumin (BSA) for 30 minutes. The samples were washed three times with DPBS between each procedure. Next, fixed samples were incubated in primary antibodies (Table S2, Supporting Information) for 2 hours at room temperature, washed with DPBS three times, and then secondary antibodies (Table S2, Supporting Information) for 1.5 hours. Finally, after three DPBS washes, the cells were incubated with DAPI for nuclei staining for 10 minutes. The bright‐field and epifluorescence microscopy was performed on a Nikon Eclipse Ti microscope with Zyla 4.2 PLUS sCMOS camera.

### Tri‐lineage Differentiation

StemPro Adipogenesis Differentiation Kit (*Life Technologies, Ca# A1007001*) was used to induce adipogenic differentiation of iMSCs at MP6. iMSCs were plated in a 12‐well plate at 1 × 10^4^ cells cm^−2^ for 4 days in serum‐free MSC culture medium (*CTS StemPro MSC SFM, Life Technologies, Ca# A1033201*). The cells were then treated with the complete adipogenesis medium consisting of adipocyte differentiation basal medium, adipogenesis supplement and gentamicin. Medium was refreshed every four days for 20 days. On Day 21, cells were processed with Oil Red O to detect lipid droplets.

StemPro Osteogenesis Differentiation Kit (*Life technologies, Ca# A1007201*) was used to induce osteogenic differentiation of iMSCs at MP6. iMSCs were plated in a 12‐well plate at 5 × 10^3^ cells cm^−2^ for 3 days in serum‐free MSC culture medium. The cells were then treated with the complete osteogenesis medium consisting of osteocyte/chondrocyte differentiation basal medium, osteogenesis supplement and gentamicin. Medium was replaced every four days for 20 days. On Day 21, cells were incubated with anti‐osteocalcin primary antibody overnight at 4 °C and then secondary antibody for 2 hours at room temperature.

StemPro Chondrogenesis Differentiation Kit (*Life Technologies, Ca# A1007101*) was used to induce chondrogenic differentiation of iMSCs at MP6. The iMSC‐contained solution of 1.6 × 10^7^ cells mL^−1^ was produced in serum‐free MSC culture medium. To create micro‐mass culture, 5 uL cell solution was transferred onto a 12‐well plate for 2 hours (4‐5 micro‐mass culture per well). The micro‐mass culture was then treated with the complete chondrogenesis medium consisting of osteocyte/chondrocyte differentiation basal medium, chondrogenesis supplement and gentamicin. The medium was refreshed every three days for 20 days. On Day 21, the micro‐mass was stained with anti‐aggrecan primary antibody overnight at 4 °C and then secondary antibody for 2 hours at room temperature.

### Flow Cytometry Analysis

Cells were singularized with 0.25% trypsin for 5 minutes and quenched with serum‐free media. After washing with DPBS three times, cells were fixed with 4% (vol/vol) paraformaldehyde (PFA) for 15 minutes, washed and incubated with fluorescent conjugated antibodies against cell surface markers: CD105, CD90, CD45 and CD73 (Table S2, Supporting Information) for 45 minutes. The labeled cells were analyzed by the BDAccuri C6TM flow cytometer at the Syracuse University Flow Core.

### Cell Sorting

To create a relatively homogenous iMSC population for RNA sequencing analysis and anti‐inflammatory function assessment, CD73+ cells were isolated based on fluorescence‐activated cell sorting (FACS). Cells were dissociated and singularized using 0.25% trypsin and centrifuged at 250 g for 10 minutes to pellet. Next, cells were washed two times by resuspending in FACS buffer (PBS with 10% fetal bovine serum (FBS)) and centrifuging into pellets. Next, cells were resuspended in the FACS buffer with CD73‐conjugated antibody (*BD Bioscience, Ca# 560847*) and incubated for one hour on ice. After incubation, cells were washed three times and resuspended in the FACS buffer for sorting. Cells were sorted on BD FACSAria II SORP (Syracuse University Flow Core) directly into TRIzol reagent (*Life Technologies, Ca#15596018*) for cell lysis and RNA extraction.

### RT‐qPCR

Total RNA was extracted and purified using RNeasy mini kit (*Qiagen Inc, Ca#. 74104*). The isolated RNA was quantified by measuring the absorbance at 260 nm and 280 nm using a NanoDrop Microvolume UV–vis Spectrophotometer. cDNA was synthesized using thermocycler per manufacturer's instructions using SuperScript IV Reverse Transcriptase (*Life technologies, Ca# 18090010*), Oligo(dT)20 primer (*Life technologies, Ca# 18418020*), dNTP Mix (*Life Technologies, Ca# 18427013*), and RNaseOUT Recombinant Ribonuclease Inhibitor (*Life Technologies, Ca# 10777019*). cDNA was diluted and aliquoted into a 96‐well customized TaqMan Array (*Life Technologies, Ca# 4391525*) containing pre‐dispensed gene specific primer sets, together with Fast Advance TaqMan Master Mix *(Life Technologies, Ca# 4444964*). The customized TaqMan array plate contained one manufacturing control gene (18S), three candidate endogenous control genes (GAPDH, HPRT, GUSB), and genes‐of‐interest with four replicates (Table S3, Supporting Information). Real‐Time quantitative PCR (qRT‐PCR) was performed using a QuantStudio 3 Real‐Time PCR System. ‐∆Ct value was calculated and averaged for Figures 1 and 2. ‐∆∆Ct was calculated relative to the negative control.

### Bulk RNA Sequencing and Bioinformatics Analysis

Total RNA was extracted from CD73+ sorted NC‐iMSCs and CT‐iMSCs, together with hiPSCs, intermediate cells (neural crest cells and cytotrophoblast cells), and primary MSCs (BM‐pMSCs, AD‐pMSCs, UC‐pMSCs, DP‐pMSCs, CP‐pMSCs and CV‐pMSCs) for standard bulk RNAseq analysis. The samples were incubated with TRIzol reagent at room temperature for 5 minutes with vortexing. The RNA was isolated using RNeasy mini kit (Qiagen Inc, Ca#. 74104), quantified using a NanoDrop Microvolume UV–vis Spectrophotometer, and stored at −80˚C. The RNA quality was evaluated using Agilent 2100 Bioanalyzer at Molecular Analysis Core, SUNY Upstate Medical University. The samples with RIN ≥ 8.0 and concentration ≥ 50 ng µL^−1^ were sent to Azenta USA Inc. for standard RNA sequencing services. The Illumina Ribo‐Zero rRNA removal kit was used for rRNA depletion of all the samples. The samples were sequenced using Illumina HiSeq with 2  × 150 bp configuration, single index, paired end reads per lane.

The raw FASTQ files were analyzed using the Partek Flow software, courtesy of a shared license provided by SUNY Upstate Medical Genomics Core. The unaligned reads were trimmed for bases to obtain a Phred quality score > 20, and then aligned using the Spliced Transcripts Alignment to a Reference (STAR) to the human genome (hg38). The post‐alignment assessment was conducted for quality assurance (QA) and quality control (QC), which showed the percentage of alignment for each sample was > 75%. The total number of reads for each sample was between 37 million to 52 million with a %GC ranging from 48.54% to 67.62%, which were within the recommended values for profiling human gene expression. The average base quality score per read was between 35.8 and 38.7, indicating good quality reads. Post‐alignment quantification was applied to an annotation model and normalized based on recommended parameters of counts per million (CPM). The downstream analysis included principal component analysis (PCA), differential gene expression (DESeq), hierarchical clustering, gene ontology (GO) and pathway analysis. Gene network analysis was performed using Cytoscape 3.9.1 with GeneMANIA library.

### Single Cell RNA Sequencing and Bioinformatics Analysis

The MP6 CT‐iMSCs and NC‐iMSCs were used for single cell RNA sequencing (scRNAseq) analysis. Upon confluence, iMSCs were harvest using 0.25% trypsin‐EDTA and resuspended in DMEM‐FBS solution. Single‐cell suspensions were counted using an automated cell counter (Chemometec NC‐200), and concentrations were adjusted to 5 × 10^5^ cells per ml. Single‐cell suspensions were processed in the Cornell BRC system by the Chromium Controller (10x Genomics) using the Chromium Next GEM Single Cell 3′ Reagent kit. Cells were diluted into the Chromium Single Cell A Chip to yield a recovery of 5000 single‐cell transcriptomes. After preparation, libraries were sequenced using a NextSeq2000 P2‐100 (90 nt cDNA read), ≈400 M reads.

The raw FASTQ files were analyzed using the Partek Flow software. The unaligned reads were trimmed, aligned using STAR 2.7.8a with homo sapiens (human) – hg38, processed with UMI deduplication, filtered and quantified the barcodes based on annotation model of Ensembl Transcripts release 100, which produced the single cell count matrices for downstream analysis. Further quality control and preprocessing were performed on each sample individually. Cells with fewer than 500 features detected or fewer than 1500 unique molecules detected were removed. Cells with more than 15% of unique molecules mapping to the mitochondrial genome were removed. Features detected in less than 5% of the cells were removed. After these preprocessing and quality control, 2223 CT‐iMSCs and 2865 NC‐iMSCs were retained with total 13122 genes for subsequent analysis.

After quality control, samples were then merged. Gene expression data was normalized, log‐transformed, and scaled. Principal component analysis was first conducted, and resulted embedding was analyzed using graph‐based Louvain clustering algorithm with a resolution of 0.5 and number of nearest neighbors of 30. The resulting clusters were visualized using uniform manifold approximation and projection (UMAP), and then labeled and annotated according to a set of curated canonical gene markers. Next, top 1000 most variable genes were identified and used for hierarchical clustering, as well as gene set and pathway enrichment analysis. Trajectory pseudo‐time analysis was performed using Monocle 3 algorithm with attribute value for root nodes of 1.

### Small Extracellular Vesicle (sEV) Purification

iMSCs were plated at a density of 2.5 × 10^4^ cells cm^−2^ in the serum‐free MSC culture media (*CTS StemPro MSC SFM, Life Technologies, Ca# A1033201*) on 6‐well plates coated with 1% Gelatin solution. After 3 days, the iMSCs were primed by either 2 µL/mL of Lipopolysaccharide (LPS) solution (500X, *Life technologies, Ca# 00‐4976‐03*) or cell stimulation cocktail (CSC, containing phorbol 12‐myristate 13‐acetate (PMA), ionomycin, brefeldin A and monensin) solution (500X, *Life Technologies, Ca# 00‐4970‐03*) for three days. The media was collected and filtered by vacuum filtration to remove residual cells, debris and large particles for EV isolation and purification.

Filtered primed cell supernatant media was concentrated to remove excess water using Amicon 100 kDa ultracentrifugation filters at 4000 × g for 20 minutes. The concentrated samples were moved to a clean sterile tube. The filtrate was centrifuged twice at 4000 × g for 10 minutes to recover any additional particles that may have flowed through during initial centrifugation. The samples were collected and incubated with 0.5 volumes of Total Exosome Isolation Reagent (*Life Technologies, Ca# 4478359*) per manufacturer's instruction. The suspension was vortexed thoroughly to form a homogenous solution, and then incubated overnight in the refrigerator. Next day, samples were centrifuged at 2˚C, 10000 × g for an hour and discarded the supernatant. The pellet at the bottom of the tube was resuspended in sterile 1X DPBS, and further filtered by size exclusion chromatography using qEV columns (*qEVoriginal/35 nm Gen 2 Column, IZON Inc*.) with an optimum recovery range of 35 nm to 350 nm.

### Nanoparticle Tracking Analysis (NTA)

NTA was performed to estimate the concentration and size distribution of sEVs collected from primed iMSCs. For each run, 300 µL of the prepared sEV samples were injected into the sample chamber of a NS300 instrument (*NanoSight, Aumesbery, UK*) with a 532 nm green laser. Seven measurements of each sample were performed for 30 seconds each. The default adjustment settings (Blur, Minimum expected particle size, and Minimal track lengths) provided by the software were used. The camera level (9–12) and detection threshold (2–6) were adjusted manually for each experiment as recommended by the manufacturer. For data capturing and analysis, the NTA analytical software (*NanoSight NTA version 3.2*) was used. Briefly, from the recorded video, the mean square displacement of each detected particle was determined. Then, using the Stokes‐Einstein equation, the diffusion coefficient and sphere‐equivalent hydrodynamic radius were determined by the software.

### Western Blot

Total protein concentrations from the EVs determined by the BCA micro assay kit (*Cat. #: 23235, Thermo Scientific*). 15 µg of protein were separated by SDS‐PAGE gel, then transferred to PVDF membranes (*Cat. #: IPVH00010, Millipore Co., Ltd*). The membranes were incubated with 5% non‐fat milk (*Cat. #: NC9952266, Fisher Scientific*) in Tris‐buffered saline plus 0.5% Tween‐20 for 1 hour at room temperature, and then overnight at 4˚C with primary antibodies (Table S2, Supporting Information). The secondary antibody linked to horseradish peroxidase (HRP) purchased from Santa Cruz Biotechnology (*Cat. #: 516102, 1:1000 dilution*) was applied for 1 hour at room temperature. Antibody‐antigen complexes were visualized using ECL (*Cat. #: 34580, Thermo Scientific*) according to the manufacturer's instructions.

### Luminex Multiplexing Assay

sEVs were characterized based on multiplexed Luminex assays using Exosome Characterization 6‐Plex Human ProcartaPlex Panel [CD9, CD63, CD81, Cytochrome C, Syntenin‐1, VLA‐4] (*Invitrogen, Ca#: EPX060‐15845‐901*) and Cytokine 10‐Plex Human Panel [GM‐CSF, IFNγ, IL1β, IL2, IL4, IL5, IL6, IL8, IL10, TNFα] (*Invitrogen, Ca#: LHC0001M*) per manufacturer's instructions. sEV suspension was centrifuged at 10000 × g for one hour into pellet, which was lysed with Exosome Resuspension Buffer (*Life technologies, Ca#4478545*) and stored at −20˚C until further analysis. Standards were prepared from supplied lyophilized standard mix, reconstituted, and diluted with the 1X wash buffer in a serial dilution. Capture bead mix was added to the plate and washed. The standards and samples were added to assigned wells, sealed at room temperature for 2 hours with shaking at 600 RPM. The wells were washed three times before adding the Biotinylated detection antibody. Next, the plate was sealed and shaken at 600 RPM for 30 minutes. After washing three times, Streptavidin‐PE‐(SA‐PE) was added to each well, and the plate was sealed and shaken at room temperature for 30 minutes. After washing three times, the reading buffer was added, and the plate was sealed and shaken for 5 minutes. The plates were run on a BioPLex 200 xMAP instrument in the Genomics Core at SUNY Upstate Medical University. The detection limit was set as 100 pg mL^−1^. The concentration lower than the detection limit was counted as Not Detected (ND), and value was used as “0” in the statistical analysis.

### Statistical Analysis

All the statistical analysis was completed in Prism 9 software. Data was plotted as box plots or mean ± s.d. For single comparisons between two individual groups, a two‐sided Student's t‐test was used, and p ≤ 0.05 was considered significant. For comparisons between more than two groups, analysis of variance (ANOVA) was performed and p ≤ 0.05 was considered significant. ANOVA analysis was supplemented with post‐hoc Tukey's multiple comparison tests to determine significance between groups.

## Conflict of Interest

The authors declare no conflict of interest.

## Author Contributions

T.W. and Y.S. contributed equally to this work. T.W. Y.S. and Z.M. designed the experiments. W.T. and Y.S. performed the biological experiments and data analyses. J.Y. performed mesenchymal stem cell culture and extracellular vesicles collection. Y.S. and H.S. performed bioinformatics analysis on the bulk RNA sequencing data. Z.M. performed bioinformatics analysis on single‐cell RNA sequencing data. M.A. and T.R.G. performed the nanoparticle tracking analysis on the extracellular vesicles. M.I.K. provided the Yale hiPSC line. Y.S. and Y.W. designed the co‐culture experiments between RAW267 cells and mesenchymal stem cells. Q.M. and R.N.G. performed the protein analysis on the extracellular vesicles. T.W., Y.S., and Z.M. wrote the manuscript with discussion and improvement from all the authors. Z.M. supervised the project development and funded the study.

## Supporting information

Supporting Information

## Data Availability

The data that support the findings of this study are available from the corresponding author upon reasonable request.
