# Supplementary material for: Lineage‐Specific Mesenchymal Stromal Cells Derived from Human iPSCs Showed Distinct Patterns in Transcriptomic Profile and Extracellular Vesicle Production
Source: Adv Sci (Weinh). 2024 May 17;11(28):2308975. doi: 10.1002/advs.202308975 (PMC11267277; doi:10.1002/advs.202308975)
Supplement: Supplementary file 1 — Supporting Information [file ADVS-11-2308975-s001.pdf]

ADVANCED SCIENCE
Open Access

Supporting Information

for *Adv. Sci.*, DOI 10.1002/advs.202308975

Lineage-Specific Mesenchymal Stromal Cells Derived from Human iPSCs Showed Distinct Patterns in Transcriptomic Profile and Extracellular Vesicle Production

*Tackla Winston, Yuanhui Song, Huaiyu Shi, Junhui Yang, Munther Alsudais, Maria I. Kontaridis, Yaoying Wu, Thomas R. Gaborski, Qinghe Meng, Robert N. Cooney and Zhen Ma**

SUPPLEMENTAL INFORMATION

Lineage-Specific Mesenchymal Stromal Cells Derived from Human iPSCs Showed Distinct Patterns in Transcriptomic Profile and Extracellular Vesicle Production

Tackla Winston[1,2¶], Yuanhui Song[1,2¶], Huaiyu Shi[1,2], Junhui Yang[1,2], Munther Alsudais[3], Maria I. Kontaridis[4,5,6], Yaoying Wu[1,2,7], Thomas R. Gaborski[3], Qinghe Meng[8,9], Robert N. Cooney[8,9], Zhen Ma[1,2,10] *

[1]Department of Biomedical & Chemical Engineering, Syracuse University, Syracuse, NY, USA
[2]BioInspired Institute for Materials and Living Systems, Syracuse University, Syracuse, NY, USA
[3]Departments of Biomedical and Chemical Engineering, Rochester Institute of Technology, Rochester, NY, USA
[4]Department of Biomedical Research and Translational Medicine, Masonic Medical Research Institute, Utica, NY, USA
[5]Department of Medicine, Division of Cardiology, Beth Israel Deaconess Medical Center, Harvard Medical School, Boston, MA, USA
[6]Department of Biological Chemistry and Molecular Pharmacology, Harvard Medical School, Boston, MA, USA
[7]Department of Microbiology & Immunology, SUNY Upstate Medical University, Syracuse, NY, USA
[8]Department of Surgery, State University of New York Upstate Medical University, Syracuse, NY, USA
[9]Sepsis Interdisciplinary Research Center, State University of New York Upstate Medical University, Syracuse, NY, USA
[10]Department of Biology, Syracuse University, Syracuse, NY, USA

[¶]These two authors contributed equally to this work.

*Corresponding author: Zhen Ma (zma112@syr.edu)

Table S1. Primary MSCs used in this study.

Primary MSCs	*Vendor*	*Donor #*	*Lot #*
BM-pMSC	POIETICS (Lonza)	36670	18Tl282222
DP-pMSCs	POIETICS (Lonza)	37665/37664	19TL080921/19TL080920
AD-pMSCs	POIETICS (Lonza)	36295	18TL212639
CV-pMSCs	JangoCell	Human female	1911-00018-61
CP-pMSCs	JangoCell	Human female	1911-00015-31
UC-pMSCs	JangoCell	Human female	1911-00016-32

Table S2. Antibodies used for immunostaining, flow cytometry, and western blot.

Primary Antibodies	***Vendors***	***Catalog #***	***Dilution***
CD44 (mouse)	Bio-Rad Laboratories	MCA2726	1:100
CD73 (rabbit)	Abcam	ab133582	1:100
CD90 (mouse)	Abcam	ab181469	1:100
CD105 (mouse)	Life Technologies	MA5-17041	1:200
CD166 (mouse)	Bio-Rad Laboratories	MCA1926	1:100
NGFR (mouse)	Life Technologies	MA513314	1:200
SOX10 (mouse)	R&D Systems	MAB2864-SP	10μg/mL
FOXD3	R&D Systems	AF5090-SP	10μg/mL
ETS-1 (rabbit)	Life Technologies	PA581170	1:200
SNAI2 (rabbit)	Life Technologies	PA573015	1:100
KRT-19 (mouse)	Life Technologies	MA512663	2μg/mL
CDX-2 (rabbit)	Abcam	ab76541	1:500
TEAD-4 (rabbit)	Life Technologies	PA521977	1:300
GATA-3	Life Technologies	14-9966-82	5μg/mL
EPCAM (mouse)	Life Technologies	14-9326-82	5μg/mL
KRT-7 (mouse)	Abcam	AB9021	1:250
NANOG (mouse)	Life Technologies	MA1-017	1:100
SOX-2 (rabbit)	Life Technologies	PA1-094	1:200
OCT-4 (mouse)	STEMCELL Technologies	60093	1:200
Secondary antibodies	***Vendors***	***Catalog #***	***Dilution***
Alexa Fluor 488 goat anti-mouse IgG	Life technologies	A-11029	1:200
Alexa Fluor 546 goat anti-mouse	Life technologies	A11003	1:200
Alexa Fluor 488 goat anti-rabbit IgG	Life technologies	A11008	1:200
Alexa Fluor 546 goat anti-rabbit	Life technologies	A11010	1:200
Donkey Anti-Goat IgG Northern Lights NL557-conjugated	R&D Systems	NL001	1:200
Donkey Anti-Mouse IgG Northern Lights NL557-conjugate	R&D Systems	NL007	1:200
Conjugated antibodies for flow cytometry	***Vendors***	***Catalog #***	***Dilution***
CD73	BD Bioscience	560847	10 μg/ml
CD90	BD Bioscience	559869	10 μg/ml
CD105	BD Bioscience	561443	10 μg/ml
CD45	BD Bioscience	555482	10 μg/ml
Isotype FITC	BD Bioscience	555748	10 μg/ml
Isotype APC	BD Bioscience	554681	10 μg/ml
Primary antibodies for western blot	***Venders***	***Catalog #***	***Dilution***
CD9	Santa Cruz Biotech	sc-166029	1:200
CD63	Santa Cruz Biotech	sc-5275	1:200
CD81	Santa Cruz Biotech	sc-7637	1:200
HSP90α/β	Santa Cruz Biotech	sc-13119	1:200

Table S3. TaqMan PCR primers for gene expression analysis

Taqman Assay ID Gene	*Name*	*Taqman Assay ID Gene*	*Name*
Hs99999901_s1	18s rRNA	Hs00927557_m1	ETV5
Hs99999905_m	GAPDH	Hs00846583_s1	SOX11
Hs99999909_m1	HPRT	Hs00212860_m1	FOXP1
Hs99999908_m1	GUSB	Hs04189704_m1	PTPRC
Hs02387400_g1	NANOG	Hs02576480_m1	CD34
Hs00999632_g1	POU5F1	Hs00169122_g1	CD14
Hs01053049_s1	SOX2	Hs00559840_m1	KRT7
Hs00366918_m1	SOX10	Hs00231476_m1	TFAP2C
Hs00255287_s1	FOXD3	Hs01078080_m1	CDX2
Hs00609976_m1	NGFR	Hs00901885_m1	EPCAM
Hs00159686_m1	NT5E	Hs00231119_m1	GATA2
Hs00174816_m1	THY1	Hs00231122_m1	GATA3
Hs00923996_m1	ENG	Hs01125032_m1	TEAD4
Hs01075864_m1	CD44	Hs01029413_m1	TFAP2A
Hs00977641_m1	ALCAM	Hs01574644_m1	PODXL
Hs01003372_m	VCAM1	Hs00985275_g1	CGA
Hs00174838_m	MCAM	Hs00365950_g1	HLA-G
Hs00174265_m1	ANPEP	Hs00161904_m1	SNAI2
Mm99999915_g1	GAPDH	Mm00439614_m1	IL10
Mm00446190_m1	IL6	Mm00475988_m1	ARG-1
Mm00443258_m1	TNFα	Mm00434228_m1	IL1β
Hs00231692_m1	RUNX2	Hs00959010_m1	SPP1
Hs01115513_m1	PPARG	Hs01115513_m1	PPARG
Hs00153936_m1	ACAN	Hs00165814_m1	SOX9

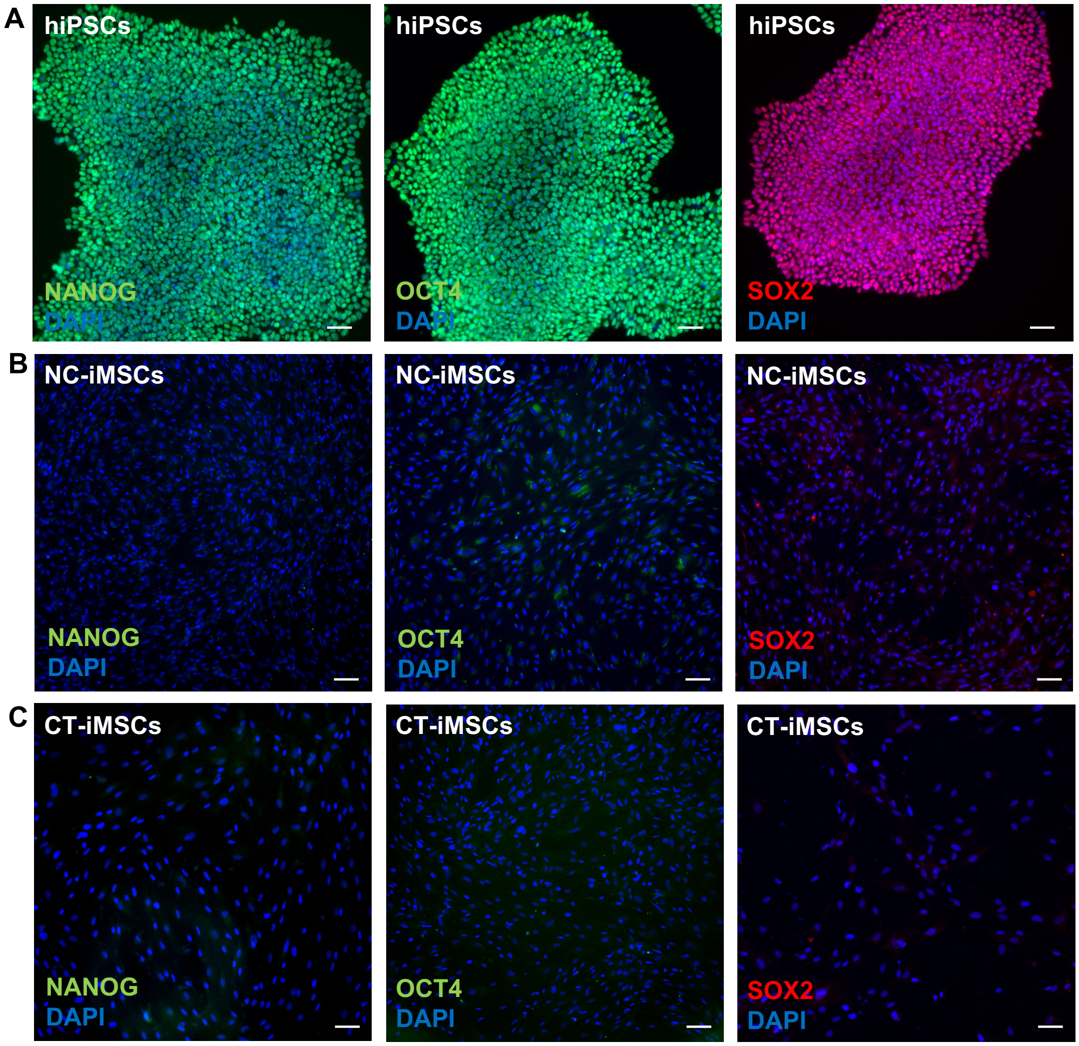

Figure S1. Immunostaining of pluripotent markers (NANOG, OCT4 and SOX2). (A) hiPSCs showed robust high expression of pluripotent markers. (B) NC-iMSCs showed very few expression of OCT4 and SOX2, while (C) CT-iMSCs showed no expression of these pluripotent markers.

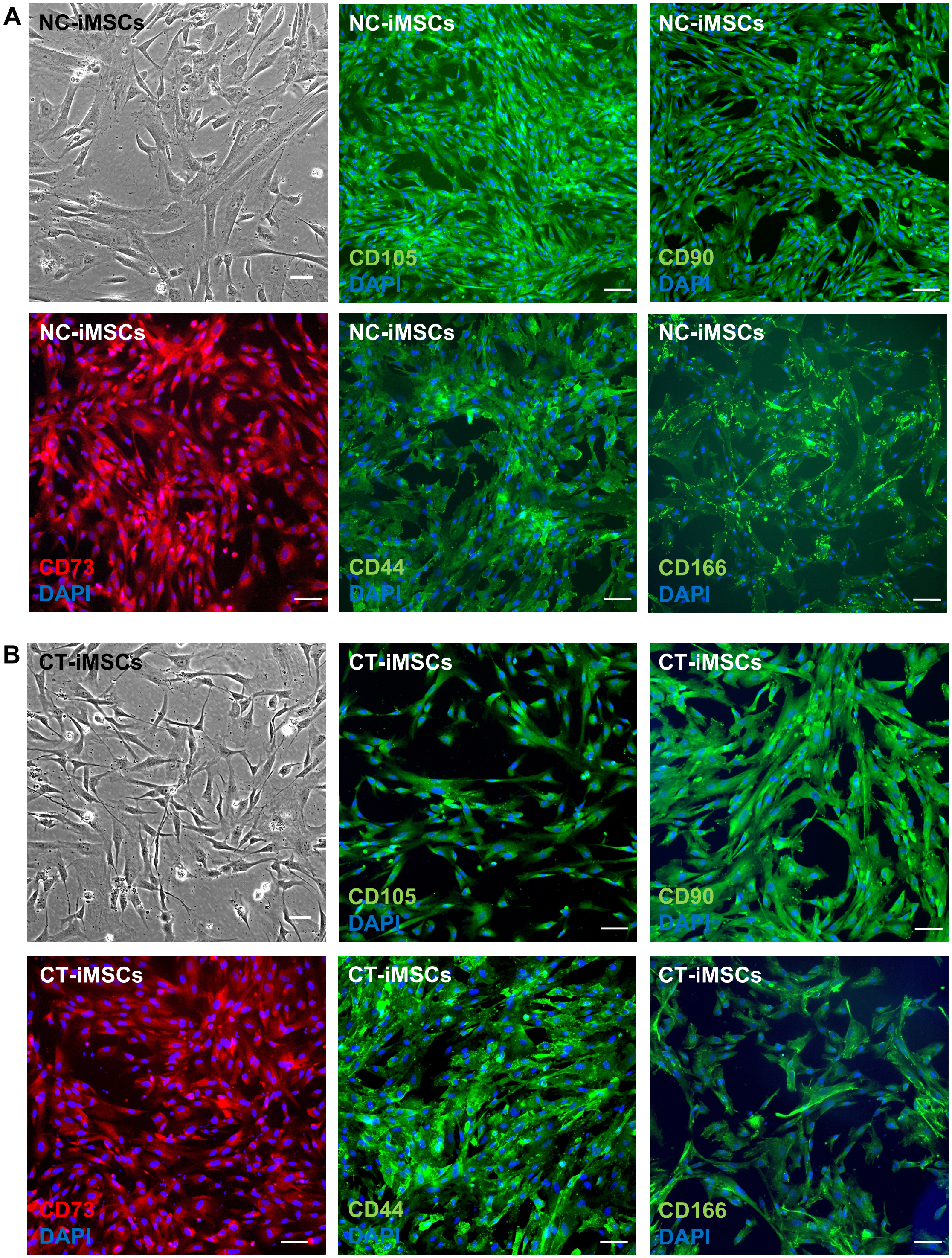

Figure S2. Differentiation of iMSCs from another hiPSC line (Yale line). Both (A) NC-iMSCs and (B) CT-iMSCs differentiated from Yale hiPSC line also showed positive staining of MSC markers (CD105, CD90, CD73, CD44 and CD166).

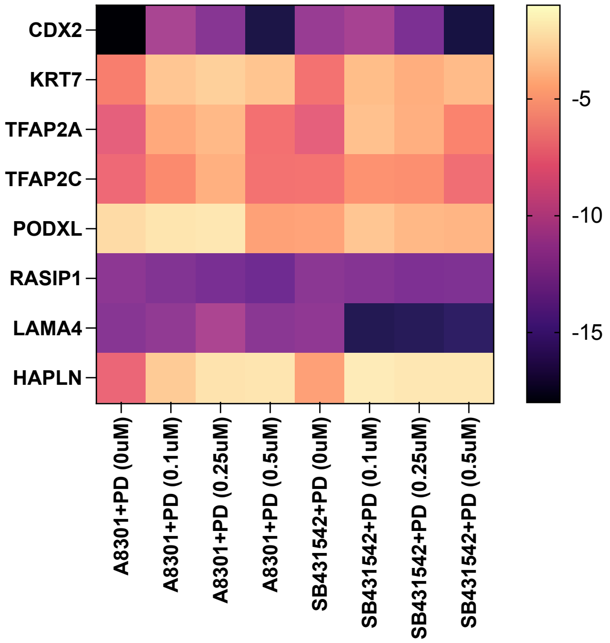

Figure S3. Optimization of intermediate cytotrophoblast differentiation. We varied the concentration of PD173074 and compared two ALK inhibitors (A8301 and SB431542) to obtain better cytotrophoblast differentiation from hiPSCs.

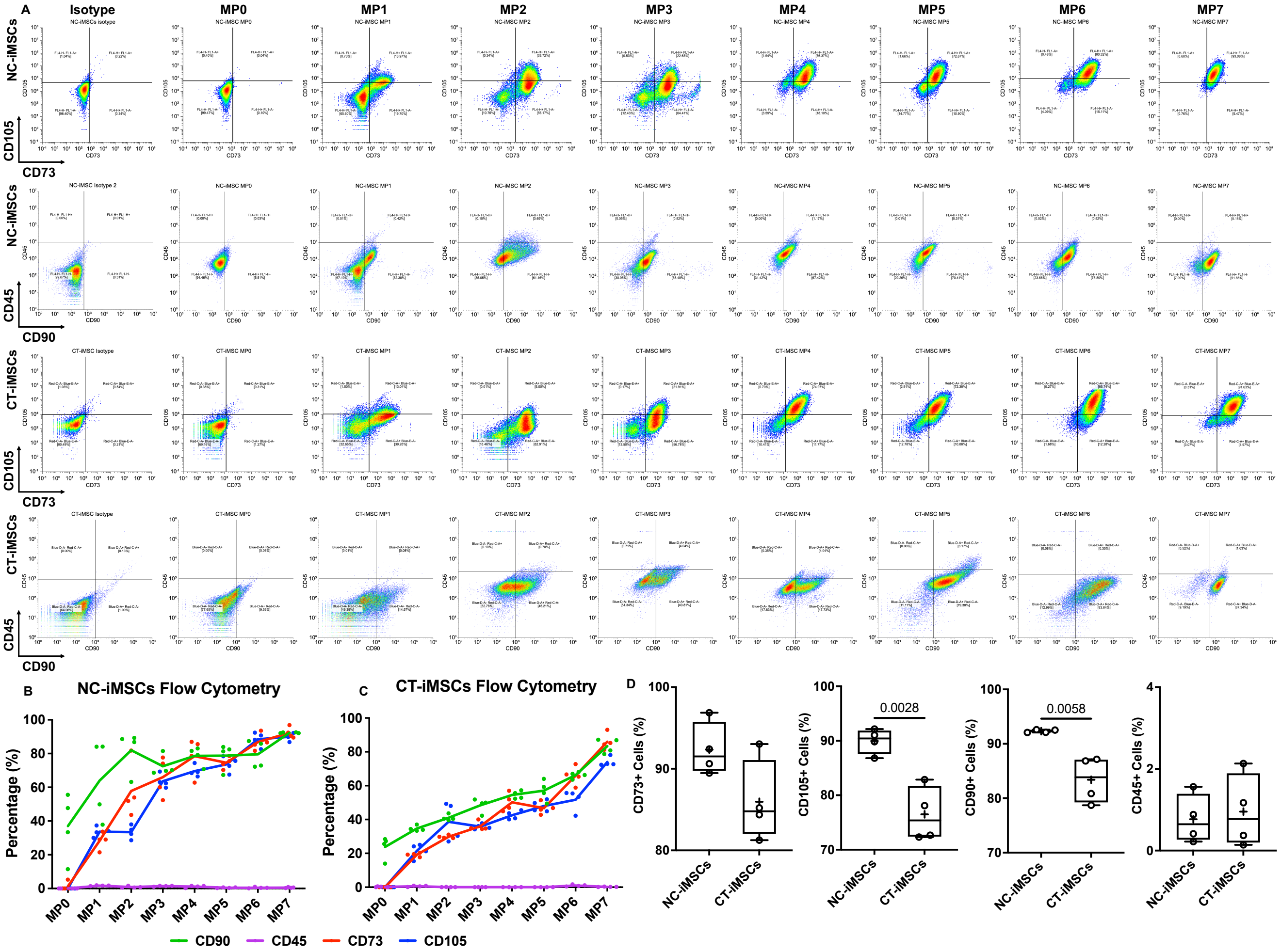

Figure S4. Emerging iMSC population during stepwise differentiation. (A) The representative density plots from flow cytometry analysis showed the emerging population of CD105+/CD73+ and CD90+/CD45- cells during iMSC differentiation. Summarized flow cytometry results showed the emerging iMSC population (B) during neural crest-to-MSC differentiation and (C) during cytotrophoblast-to-MSC differentiation. (D) NC-iMSC differentiation resulted in significantly higher percentile of CD90+ and CD105+ cells than CT-iMSC differentiation, while comparable high percentile of CD73+ cells and low percentile of CD45+ cells between two differentiations. Statistics: Student's t-test and p<0.05 is considered as significant difference (n = 4).

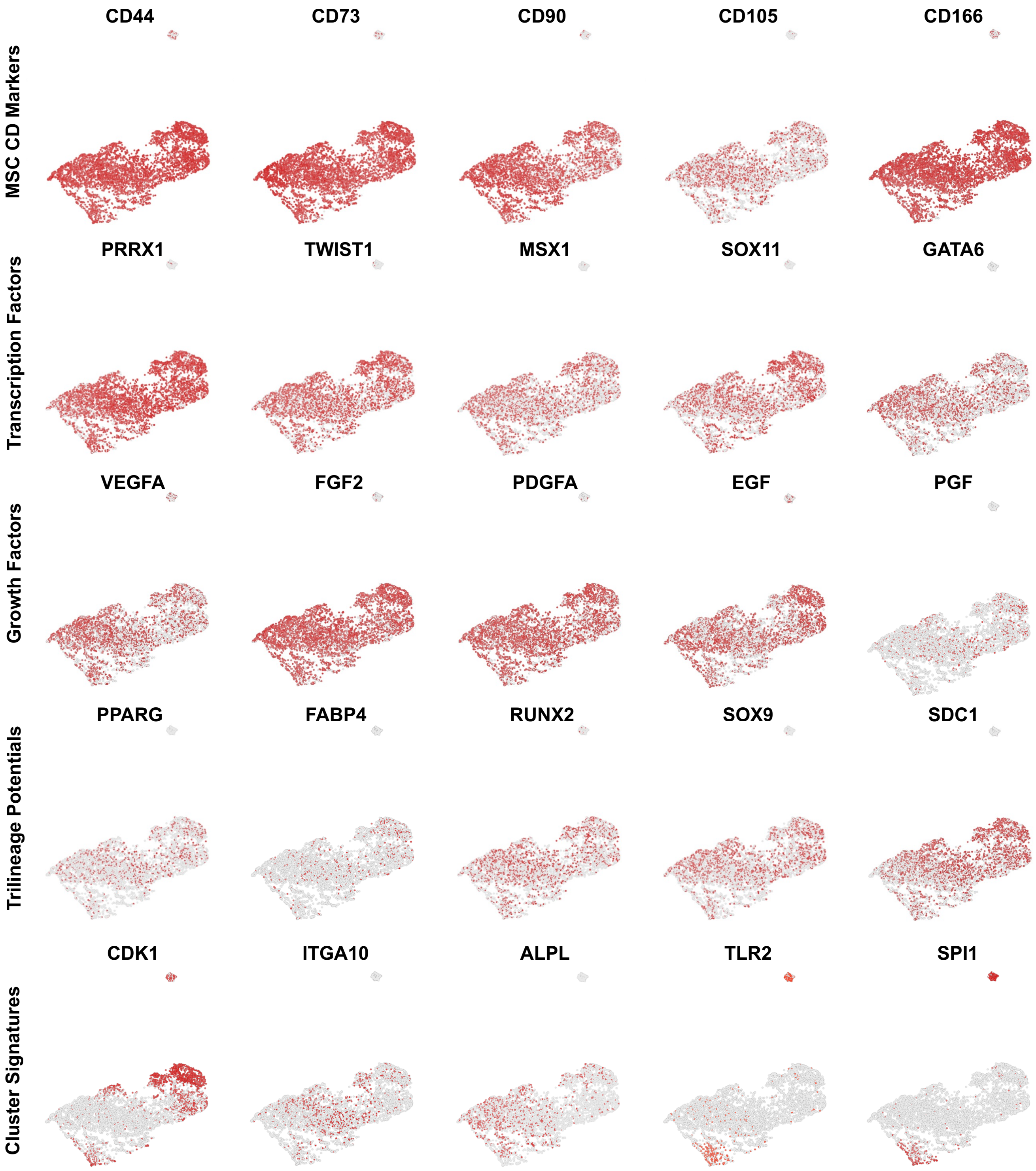

Figure S5. Single cell gene expression on UMAP projection. Visualization of single-cell gene expression associated with MSC surface markers, transcription factors, growth factors, trilineage differentiation potentials, and most variable genes for each cluster.

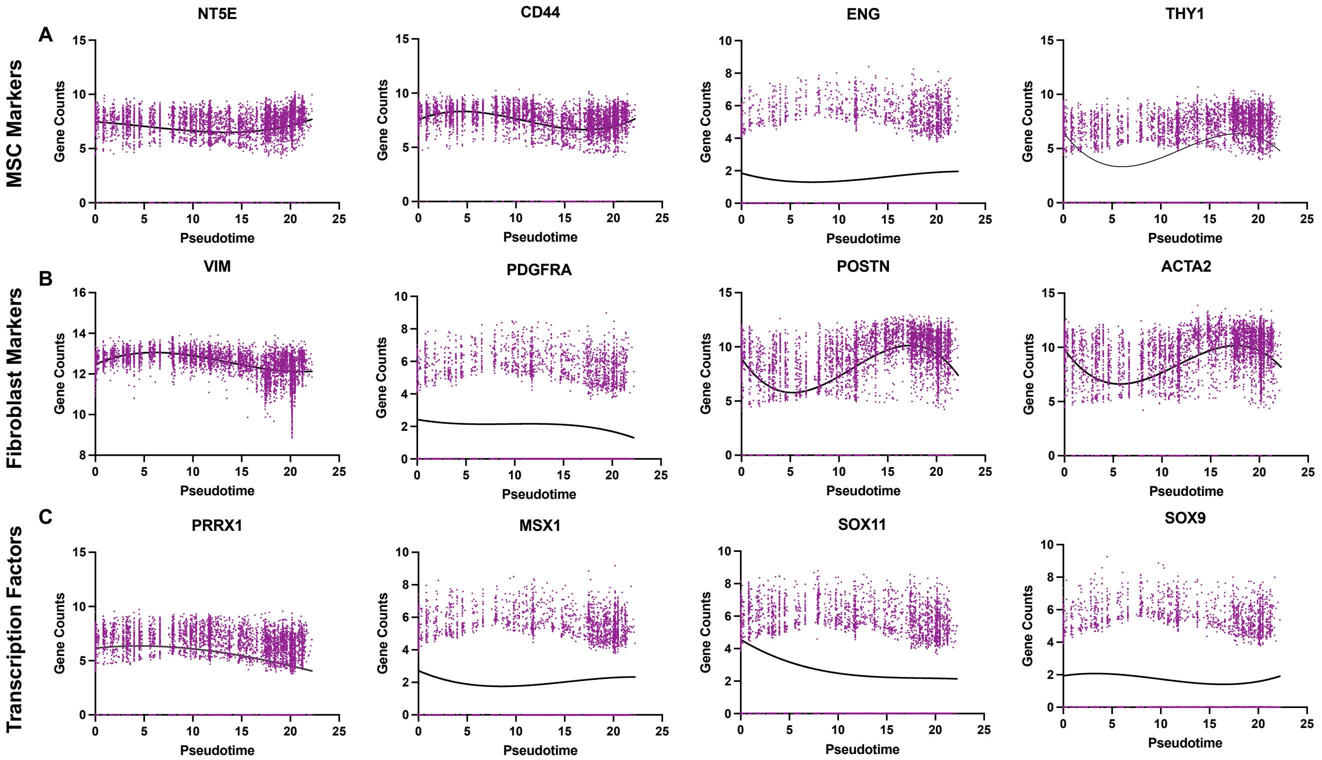

Figure S6: Gene expression across pseudo-time trajectory. Quantification of single-cell gene expression associated with (A) MSC surface markers, (B) stromal fibroblast markers, (C) and transcription factors across the entire pseudo-time trajectory of iMSC development.

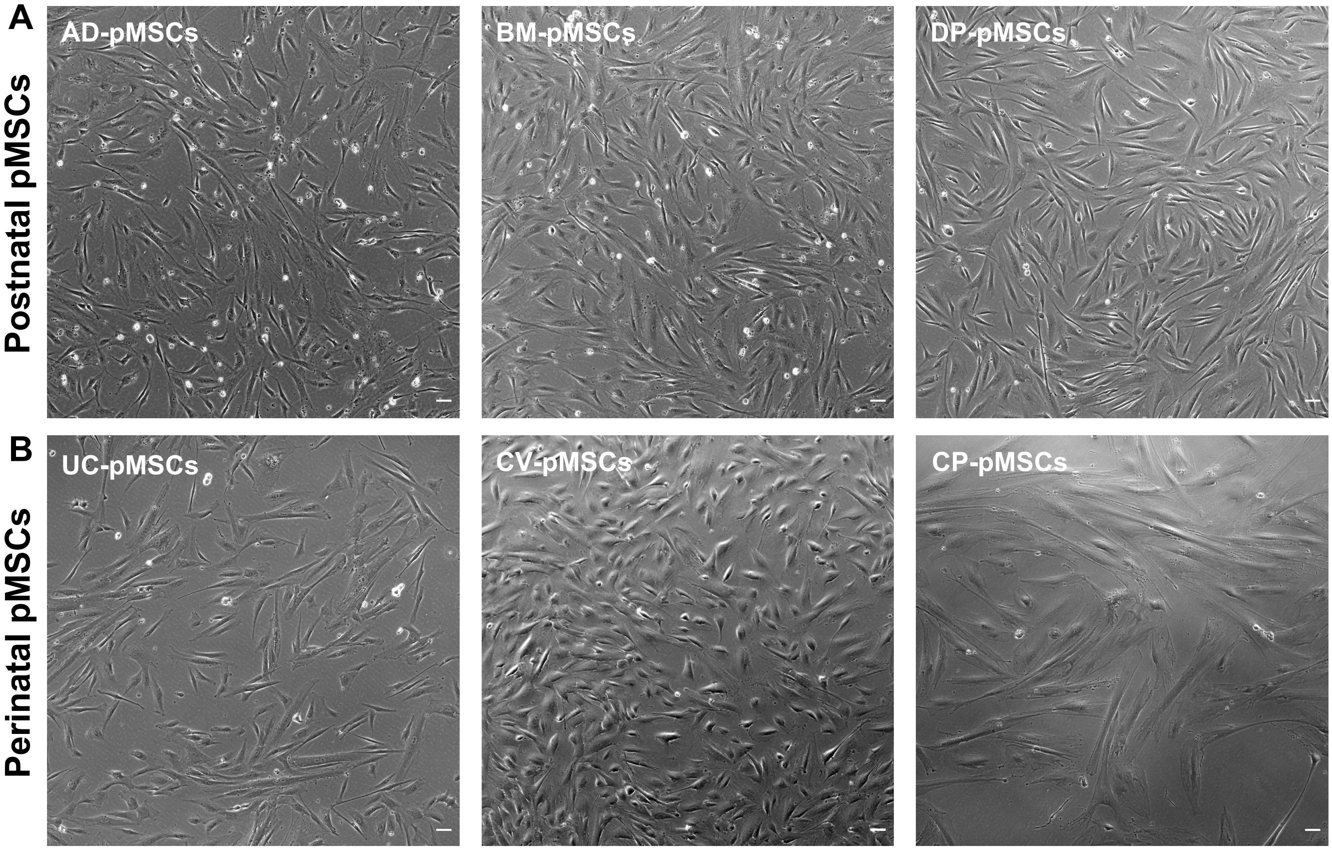

Figure S7. Phase contrast images for primary MSCs (pMSCs). (A) Postnatal pMSCs (AD-pMSCs, BM-pMSCs, DP-pMSCs) and (B) perinatal pMSCs (UC-pMSCs, CV-pMSCs, CP-pMSCs) showed spindle-like cell morphology.

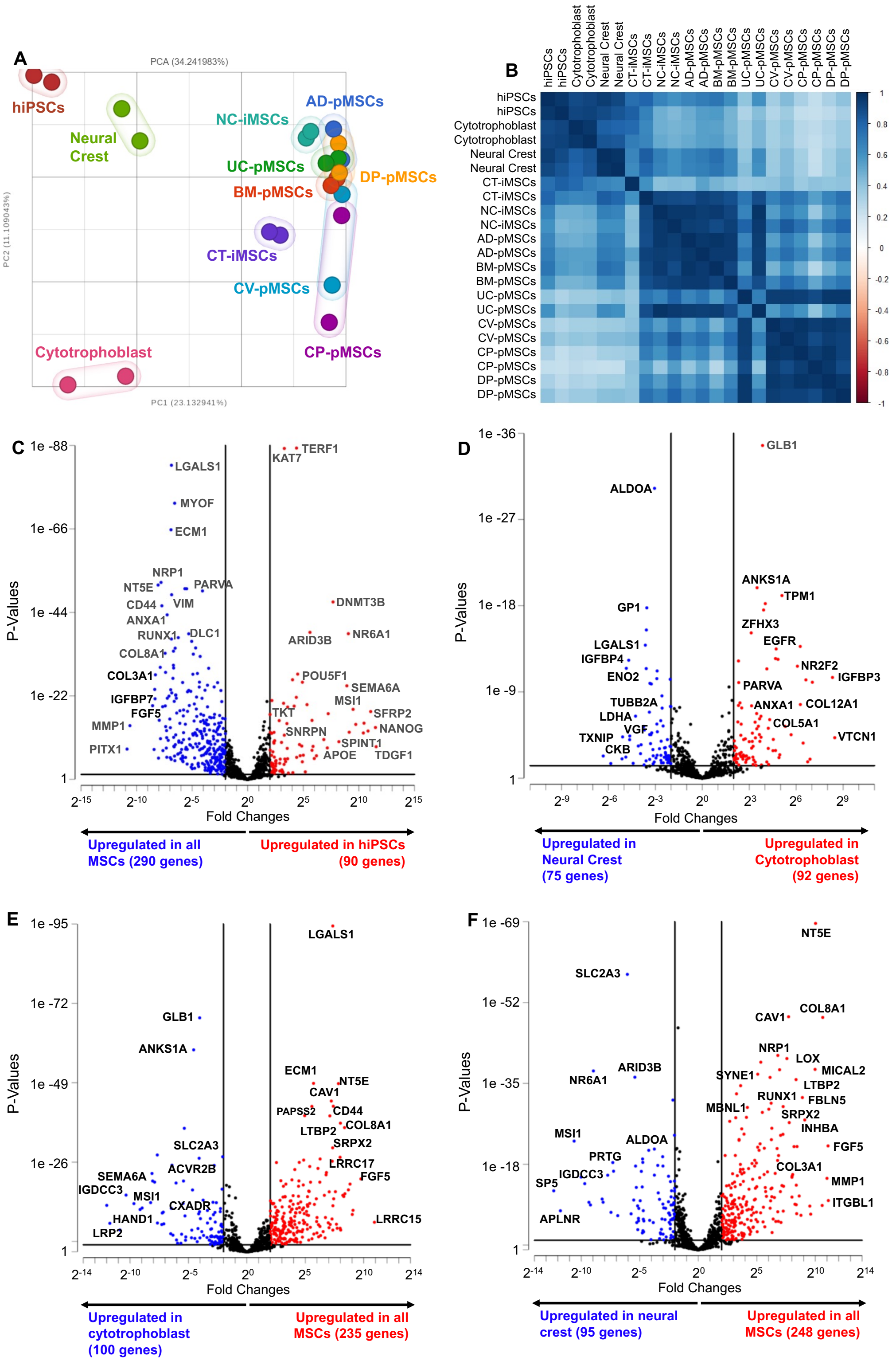

Figure S8. Transcriptomic analysis on different cell types. (A) PCA analysis and (B) Pearson's correlation matrix based on ~24,000 genes showed global relationship between hiPSCs, neural crest, cytotrophoblast, and different MSCs. Volcano plots showed differential gene expression (C) between all MSCs (6 pMSCs and 2 iMSCs) and hiPSCs, (D) between neural crest and cytotrophoblast, (E) between cytotrophoblast and all MSCs, and (F) between neural crest and all MSCs.

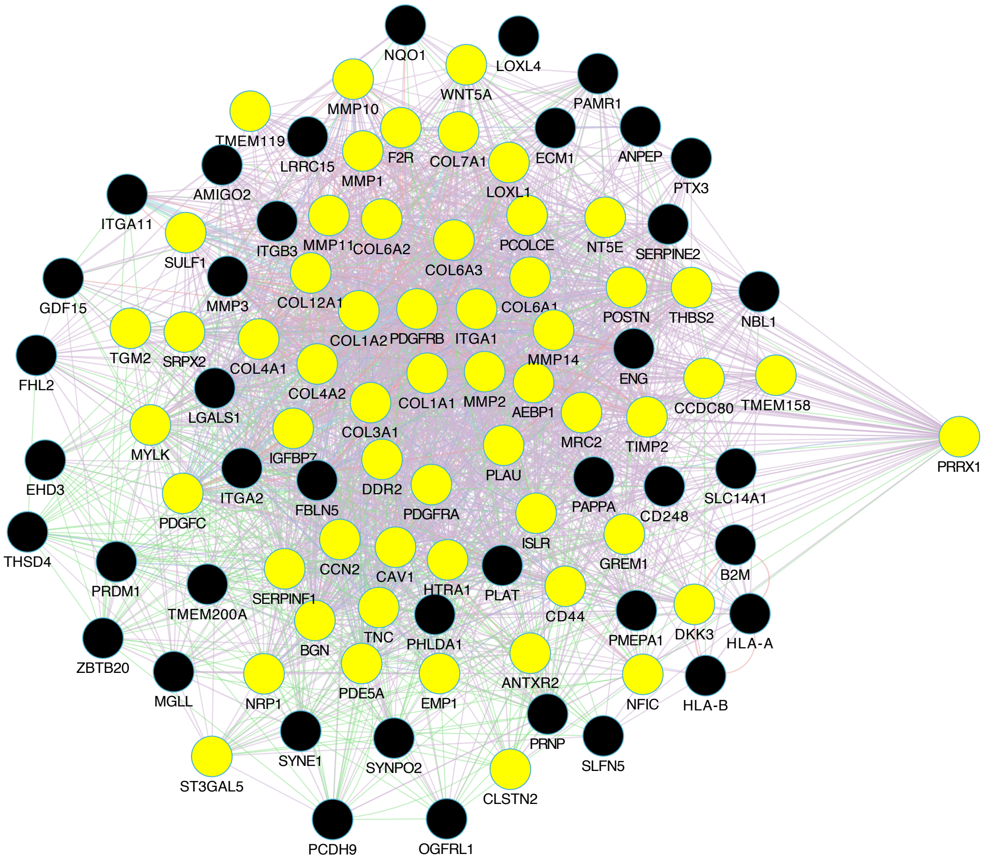

Figure S9. Network analysis on MSC-specific genes. Network analysis on upregulated genes of all MSCs compared to non-MSCs identified a key transcription factor PRRX1 associated with MSC identity (All the genes of yellow nodes are the first-neighbor connections with PRRX1).

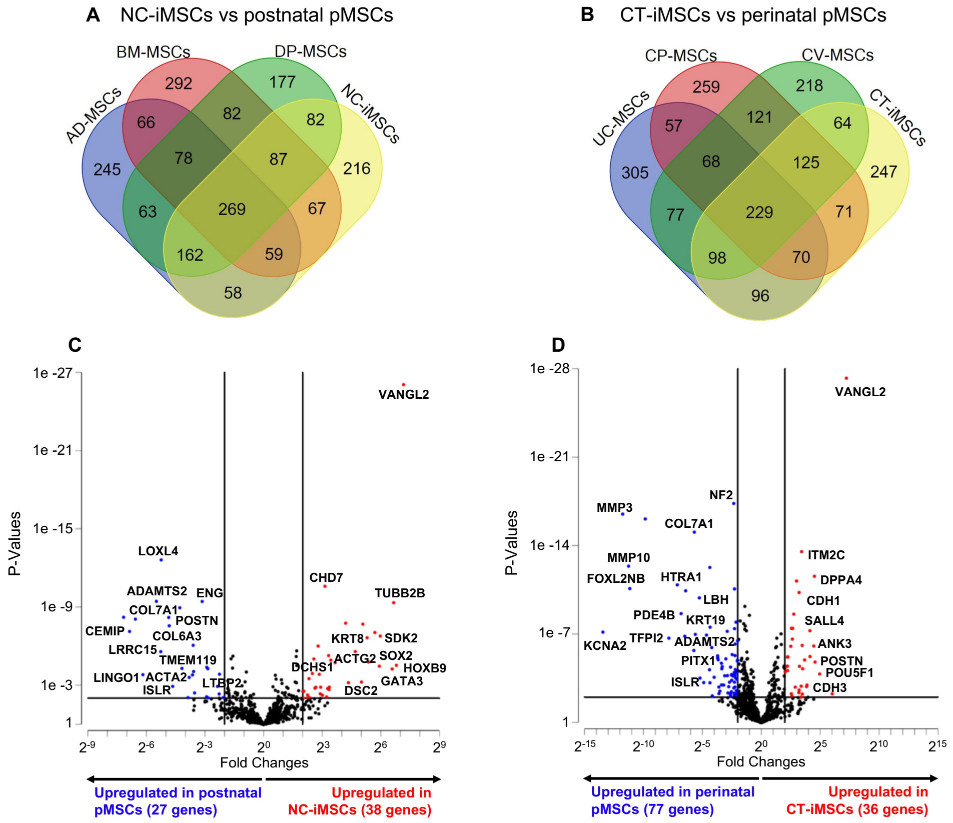

Figure S10. Transcriptomic analysis on postnatal and perinatal MSCs. (A) Four-way Venn diagrams showed transcriptomic relationship for (A) postnatal MSCs (AD-pMSCs, BM-pMSCs, DP-pMSCs, NC-iMSCs) and (B) perinatal MSCs (UC-pMSCs, CV-pMSCs, CP-pMSCs, CT-iMSCs). Volcano plots showed differential gene expression (C) between postnatal pMSCs (AD-pMSCs, BM-pMSCs, DP-pMSCs) and NC-iMSCs and (D) between perinatal pMSCs (UC-pMSCs, CV-pMSCs, CP-pMSCs) and CT-iMSCs.

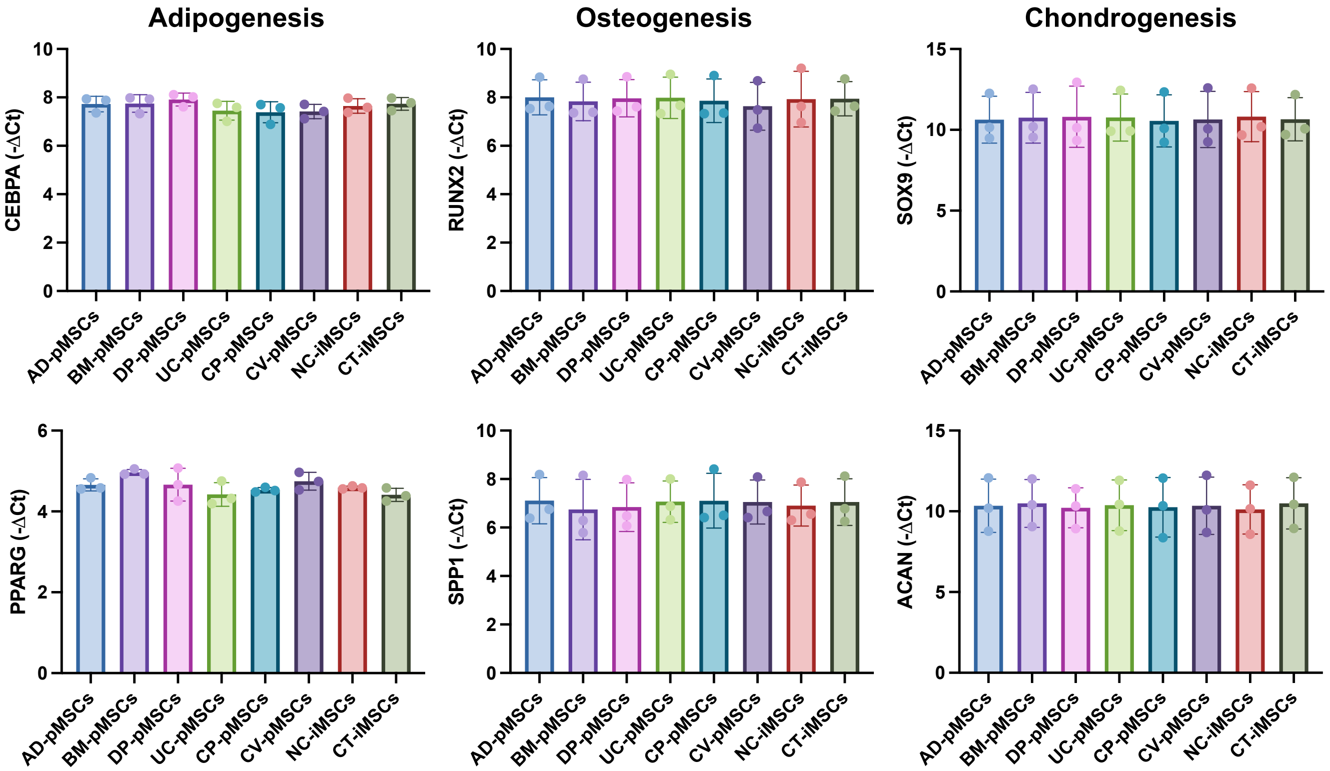

Figure S11. Tri-lineage differentiation potentials of different MSC subtypes. Tri-lineage differentiation of adipogenesis, osteogenesis, and chondrogenesis was evaluated for both NC-iMSCs and CT-iMSCs together with six different pMSC subtypes. The expression of lineage-specific genes was found no significant difference across different MSC subtypes. Statistics: one-way ANOVA with post-hoc Tukey test corrected for multiple comparison and $p<0.05$ is considered as significant difference (n = 3).

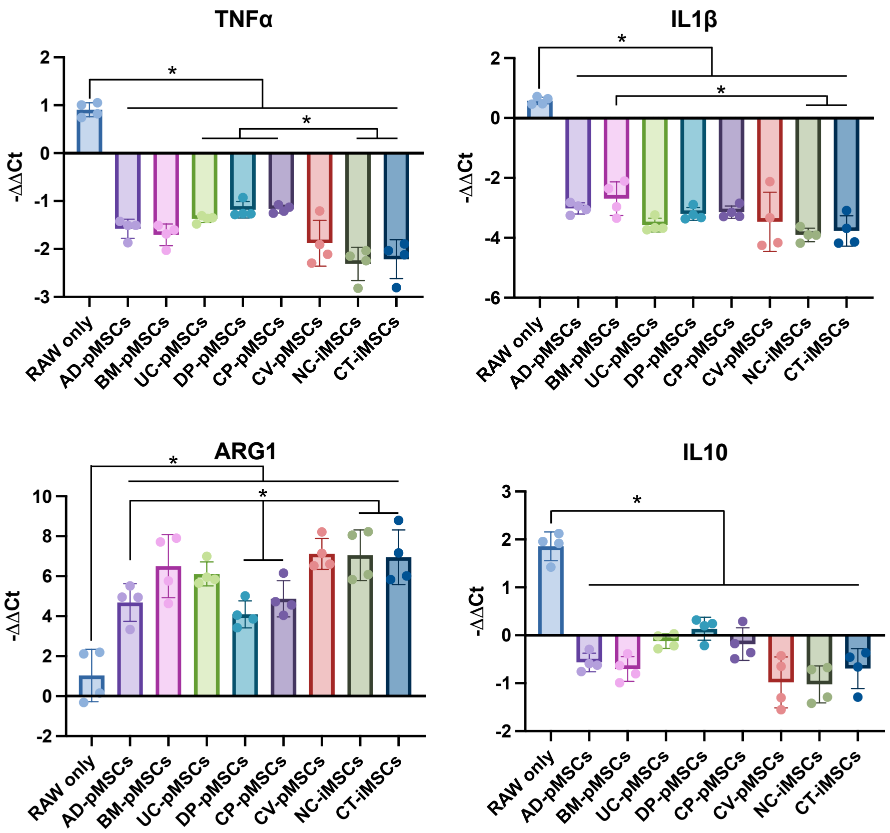

Figure S12. Anti-inflammatory function of different MSC subtypes. Co-culture with MSCs significantly attenuated cytokine gene expression (TNFα, IL1β, IL6, and IL10) but increased M2 gene expression (ARG1) of RAW264.7 cells treated with LPS. Furthermore, gene expression of TNFα and IL6 was more reduced by iMSCs (NC-iMSCs and CT-iMSCs) compared to UC-pMSCs, DP-pMSCs and CP-pMSCs. Gene expression of IL1β was more reduced by iMSCs compared to BM-pMSCs. Gene expression of ARG1 was more enhanced by iMSCs compared to AD-pMSCs, DP-pMSCs and CP-pMSCs. Statistics: one-way ANOVA with post-hoc Tukey test corrected for multiple comparison and $p<0.05$ is considered as significant difference (n = 4).

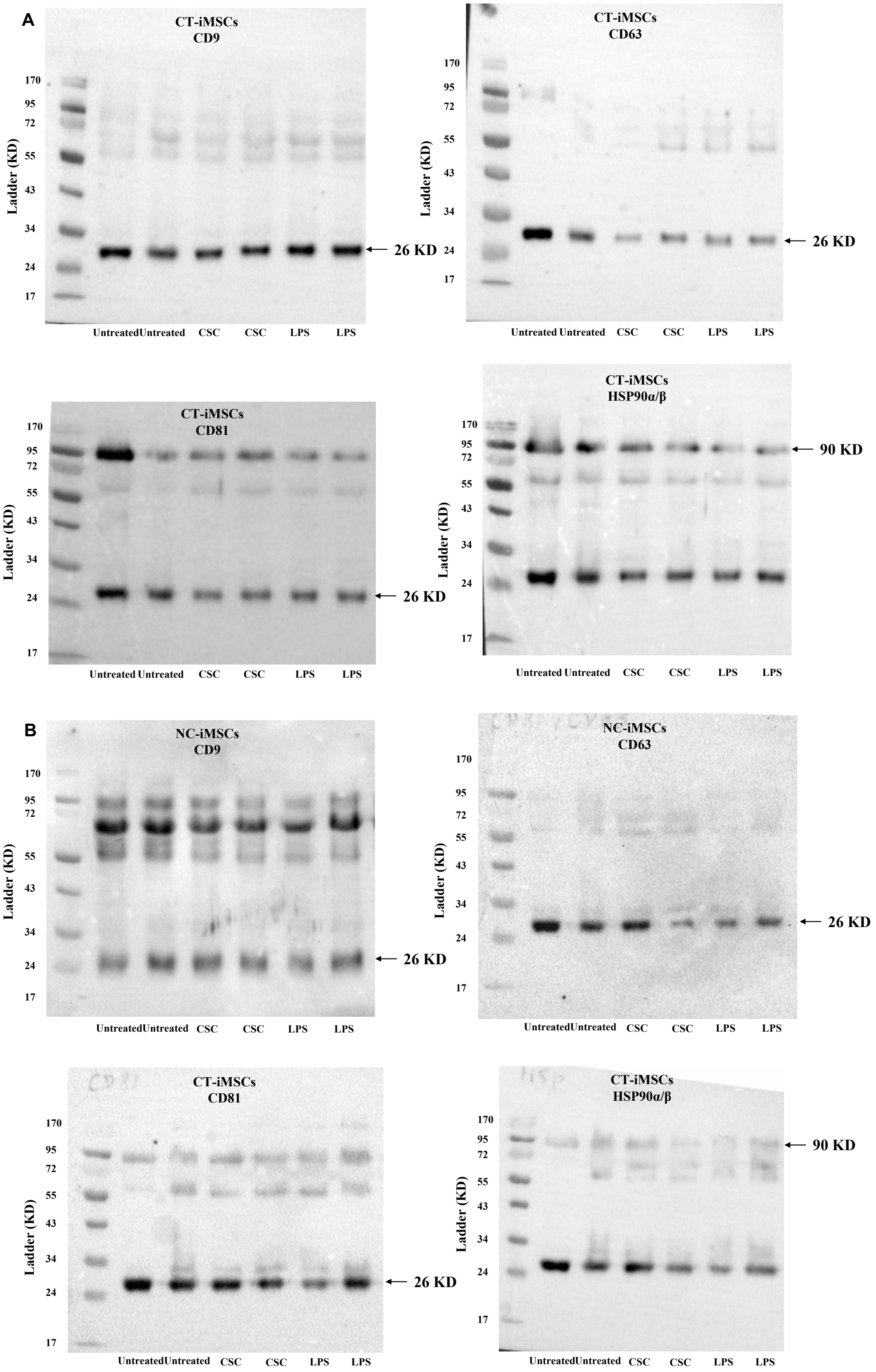

Figure S13. Raw images of western blot experiments. (A) Western blots for exosomal markers (CD9, CD63, CD81 and HSP90) for CT-iMSCs under different priming conditions. (B) Western blots for exosomal markers (CD9, CD63, CD81 and HSP90) for NC-iMSCs under different priming conditions.
